# Localisation and interactions of the Vipp1 protein in cyanobacteria

**DOI:** 10.1111/mmi.12826

**Published:** 2014-10-30

**Authors:** Samantha J Bryan, Nigel J Burroughs, Dmitriy Shevela, Jianfeng Yu, Eva Rupprecht, Lu-Ning Liu, Giulia Mastroianni, Quan Xue, Isabel Llorente-Garcia, Mark C Leake, Lutz A Eichacker, Dirk Schneider, Peter J Nixon, Conrad W Mullineaux

**Affiliations:** 1 School of Biological and Chemical Sciences, Queen Mary University of LondonMile End Road, London, E1 4NS, UK; 2 Mathematics Institute and Warwick Systems Biology Centre, University of WarwickCoventry, CV4 7AL, UK; 3 Department of Mathematics and Natural Science, University of Stavanger4036, Stavanger, Norway; 4 Department of Life Sciences, Imperial College LondonLondon, SW7 2AZ, UK; 5 Institut für Biochemie und Molekularbiologie, ZBMZ, Albert-Ludwigs-UniversitätStefan-Meier-Strasse 17, 79104, Freiburg, Germany; 6 Clarendon Laboratory, Department of Physics, University of OxfordParks Road, Oxford, OX1 3PU, UK; 7 Department of Physics and Astronomy, University College LondonGower St., London, WC1E 6BT, UK; 8 Biological Physical Sciences Institute (BPSI), Departments of Physics and Biology, University of YorkYork, YO105DD, UK; 9 Institut für Pharmazie und Biochemie, Johannes Gutenberg-Universität Mainz55128, Mainz, Germany

## Abstract

The Vipp1 protein is essential in cyanobacteria and chloroplasts for the maintenance of photosynthetic function and thylakoid membrane architecture. To investigate its mode of action we generated strains of the cyanobacteria *Synechocystis* sp. PCC6803 and *Synechococcus* sp. PCC7942 in which Vipp1 was tagged with green fluorescent protein at the C-terminus and expressed from the native chromosomal locus. There was little perturbation of function. Live-cell fluorescence imaging shows dramatic relocalisation of Vipp1 under high light. Under low light, Vipp1 is predominantly dispersed in the cytoplasm with occasional concentrations at the outer periphery of the thylakoid membranes. High light induces Vipp1 coalescence into localised puncta within minutes, with net relocation of Vipp1 to the vicinity of the cytoplasmic membrane and the thylakoid membranes. Pull-downs and mass spectrometry identify an extensive collection of proteins that are directly or indirectly associated with Vipp1 only after high-light exposure. These include not only photosynthetic and stress-related proteins but also RNA-processing, translation and protein assembly factors. This suggests that the Vipp1 puncta could be involved in protein assembly. One possibility is that Vipp1 is involved in the formation of stress-induced localised protein assembly centres, enabling enhanced protein synthesis and delivery to membranes under stress conditions.

## Introduction

Cyanobacteria and chloroplasts contain a complex internal membrane system – the thylakoid membranes – which are the site of the photosynthetic light reactions. Vipp1 (Vesicle-Inducing Protein in Plastids 1) has been implicated in thylakoid membrane biogenesis in chloroplasts (Kroll *et al*., 2001) and cyanobacteria (Westphal *et al*., 2001) based on mutant phenotypes. Vipp1 is a member of the widespread PspA/IM30 family of bacterial proteins (Westphal *et al*., 2001; Vothknecht *et al*., 2012), many of which are implicated in the maintenance of membrane integrity under stress conditions (Engl *et al*., 2009; Yamaguchi *et al*., 2013; Domínguez-Escobar *et al*., 2014). Disruption of the *vipp1* gene in *Arabidopsis* results in failure to develop normal thylakoids, and an absence of vesicles that bud from the inner chloroplast envelope membrane under certain conditions (Kroll *et al*., 2001), while disruption of *vipp1* in the cyanobacterium *Synechocystis* sp. PCC6803 results in a partially segregated mutant (*i.e*. surviving cells all retain at least one functional *vipp1* gene among their multiple copies of the chromosome) (Westphal *et al*., 2001), indicating an indispensable function for *vipp1*. The partially segregated mutant shows decreased thylakoid membrane content (Westphal *et al*., 2001). It should be noted, however, that a fully segregated *vipp1* mutant was recently produced by an indirect route in the cyanobacterium *Synechococcus* sp. PCC7002: this mutant lacks Photosystem I function but can grow heterotrophically (Zhang *et al*., 2014). Cyanobacterial *vipp1* mutants show a specific defect in Photosystem I (PSI) formation (Fuhrmann *et al*., 2009a; Zhang *et al*., 2014) and loss of *vipp1* results in loss of photosynthetic activity before the thylakoid membranes themselves are affected (Gao and Xu, 2009). This suggests that Vipp1 is more directly involved with the biogenesis of photosynthetic complexes than with the biogenesis of membranes themselves, although the two processes appear closely linked (Barthel *et al*., 2013; Zhang *et al*., 2014). In the green alga *Chlamydomonas reinhardtii,* partial depletion of Vipp1 results in a severe phenotype only under high light, and the most direct effect is on the assembly of the photosynthetic complexes, perhaps through the supply of structural lipids (Nordhues *et al*., 2012).

Vipp1 is a hydrophilic protein that shows affinity for membrane surfaces, since it can be found in thylakoid and inner envelope membrane preparations in chloroplasts (Kroll *et al*., 2001) and in thylakoid and cytoplasmic membrane preparations in cyanobacteria (Srivastava *et al*., 2005; Fuhrmann *et al*., 2009b). The first α-helical domain of Vipp1 appears to be important both for oligomerisation and for interaction with membranes (Otters *et al*., 2013). The interaction of Vipp1 with membrane surfaces is consistent with its involvement in vesicular transport between the inner envelope/cytoplasmic membrane and the thylakoids; however, direct evidence for such vesicular transport is lacking (Kroll *et al*., 2001; Vothknecht *et al*., 2012).

Immunofluorescence microscopy indicates that Vipp1 is concentrated in distinct puncta in the *Chlamydomonas* chloroplast (Nordhues *et al*., 2012). GFP-tagging reveals dynamic behaviour of Vipp1 in chloroplasts, with rapid movement of Vipp1 complexes in the envelope region, leading to the suggestion that Vipp1 plays a role in chloroplast envelope maintenance in *Arabidopsis* (Zhang *et al*., 2012). It has also been suggested that Vipp1 is required for efficient thylakoid membrane protein translocation (Lo and Theg, 2012) and that it might form a structural component of ‘thylakoid biogenesis centres’: putative localised centres of biosynthesis of thylakoid lipids and proteins in cyanobacteria and chloroplasts (Rütgers and Schroda, 2013).

Here, we use GFP-tagging and confocal microscopy to probe the dynamic behaviour of Vipp1 *in vivo* in two species of cyanobacteria. High-light exposure results in the rapid coalescence of Vipp1 into mobile puncta containing up to several hundred Vipp1–GFP molecules. Biochemical analysis of Vipp1 interaction partners *in vivo* suggests that the puncta are associated with a large and diverse collection of proteins, including a range of stress-related proteins and components of the protein synthesis and assembly machinery. This suggests a structural association between the Vipp1 bodies and sites of protein synthesis. One explanation would be that Vipp1 participates in localised protein assembly centres, required for the rapid production of new protein complexes under stress conditions.

## Results

### GFP-tagging of Vipp1 in *Synechocystis*

We used primarily the unicellular model cyanobacterium *Synechocystis* sp. PCC6803 (hereafter *Synechocystis*), which has spherical cells and rather irregular thylakoid membranes (Liberton *et al*., 2006; van de Meene *et al*., 2006). Some additional work was carried out on *Synechococcus* sp. PCC7942 (hereafter *Synechococcus*), which has elongated cells with more regular thylakoid membranes organised as concentric cylinders aligned along the long axis of the cell (Mullineaux and Sarcina, 2002). To ensure that expression of the C-terminal GFP fusions were in context and physiologically relevant, the *gfp* gene fusions were introduced into the native chromosomal *vipp1* loci (Fig. S1A). Segregation was complete (Fig. S1B), in contrast to *Synechocystis vipp1* null mutants (Westphal *et al*., 2001; Fuhrmann *et al*., 2009a; Gao and Xu, 2009). Immunoblots with anti-GFP antibody showed that GFP is linked to a protein of the expected size (Fig. S1C), while immunoblots with anti-Vipp1 antibody show that most Vipp1 is present as full-length Vipp1–GFP protein, under both low light (LL) and high light (HL) (Fig. S2A and B). The minor fragments also detected (Fig. S2A and B) are likely to be degradation products of Vipp1–GFP, resulting from the extraction process. Such degradation products were also seen in *Arabidopsis* (Zhang *et al*., 2012). Segregation of *vipp1–gfp* indicates that the GFP fusion protein retains its function. Consistent with this, *vipp1–gfp* had the same growth rate as the wild-type (not shown) and appeared phenotypically identical to the wild-type when grown under LL, with no obvious alterations in thylakoid membrane morphology (Fig. S3A–D). Since Vipp1 appears particularly important under HL (Nordhues *et al*., 2012) we also tested the tolerance of the wild-type and *vipp1–gfp* cells to HL exposure (600 μE m^−2^ s^−1^ white light for 30–60 min) by measuring light-saturated oxygen evolution before and after HL exposure. HL exposure on these timescales caused a significant decrease in oxygen evolution in all strains, indicating that photodamage was faster than repair (Fig. S3E). There was no significant difference in the light-sensitivity of oxygen evolution between *Synechocystis vipp1–gfp* and the wild-type (Fig. S3E). By contrast, a *Synechocystis* mutant deficient in the PSII repair cycle due to loss of an FtsH protease is drastically more sensitive than the wild-type to comparable HL exposure (Silva *et al*., 2003). There was no discernible effect of HL exposure on thylakoid ultrastructure, either in the wild-type or in *vipp1–gfp* (Fig. S3A–D). Since levels of Vipp1 are similar in the wild-types and the *vipp1–gfp* strains (Fig. S2A and B) it appears that the C-terminal GFP tag causes little impairment of Vipp1 function.

### Localisation and dynamics of Vipp1 in *Synechocystis*

**Figure 1:**
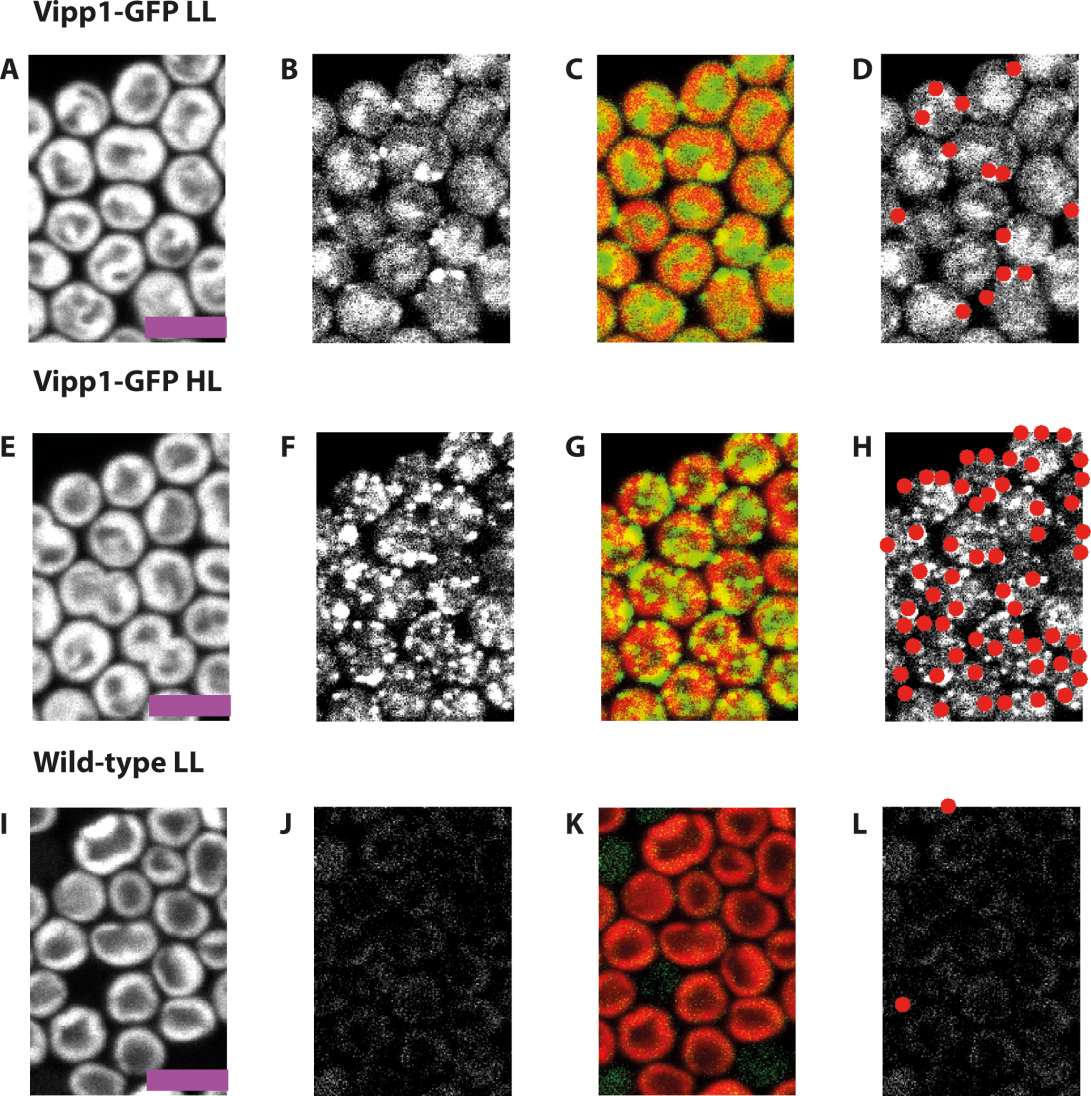
Vipp1–GFP redistributes under high-light in *Synechocystis*. Confocal fluorescence micrographs showing chlorophyll fluorescence (first column), GFP fluorescence (second column), chlorophyll (red):GFP (green) overlay (third column) and located puncta of GFP fluorescence in red (fourth column). *Vipp1–gfp* cells grown under LL (A–D) and after HL exposure for 30 min (E–H). Wild-type cells grown in LL are shown as a control (I–L). Scale bars: 5 microns.

*Synechocystis vipp1–gfp* showed strong green fluorescence under all the conditions tested (Fig. 1). To calibrate levels of background chlorophyll fluorescence, images were recorded before and after a photobleaching treatment which preferentially bleaches GFP fluorescence while having negligible effect on chlorophyll fluorescence (Spence *et al*., 2003). Under the same conditions wild-type cells showed negligible green fluorescence (Fig. 1I–L), indicating that green fluorescence gives an accurate measure of the location of Vipp1–GFP in our imaging system. Simultaneous imaging in the green and red channels allowed visualisation respectively of GFP and chlorophyll fluorescence, the latter indicating the location of the thylakoid membranes (Mullineaux and Sarcina, 2002; Spence *et al*., 2003).

**Figure 2:**
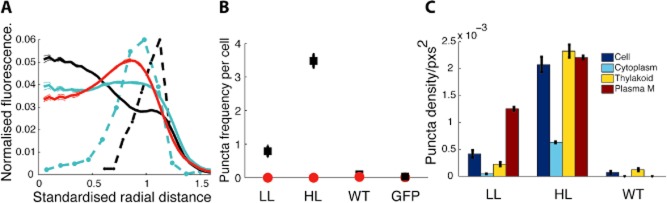
Vipp1 localisation and patterning in *Synechocystis*.A. Relative radial distribution of Vipp1–GFP fluorescence (solid, black LL; cyan HL) with S.E.M. (within line width in most cases) and puncta histogram (dashed, black, LL; cyan, HL, arbitrary vertical scale) relative to chlorophyll fluorescence (red). Standardised radial distance refers to rescaling the ½ maximum radius for chlorophyll to a radial distance of 1.B. Average counts of puncta per cell in *vipp1–gfp* cells LL, HL; WT (HL) and free GFP (HL), after bleaching in red, with S.E.M. (within marker size in most cases).C. Density of puncta (in cell, cytoplasm, thylakoid, near to plasma membrane) with S.E.M. Data from 108–370 cells from six replicate images.

**Figure 3:**
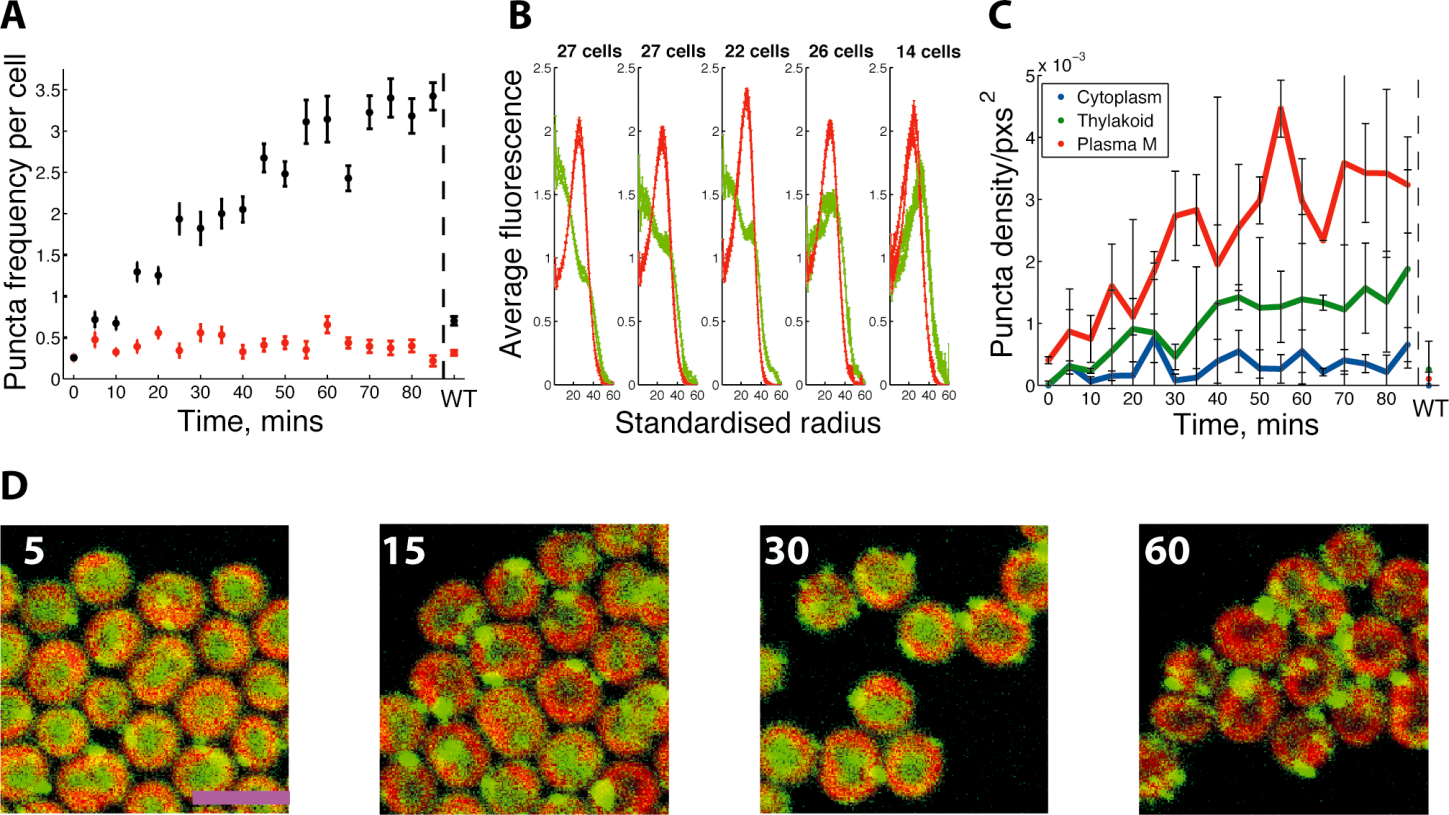
Vipp1 redistribution dynamics during HL exposure in *Synechocystis*.A. Increase in Vipp1–GFP puncta per cell during HL exposure of LL-grown *Synechocystis vipp1–gfp* for the indicated times, with S.E.M. Red points are puncta counts after bleaching to reduce GFP fluorescence. Data from WT (LL) are shown at the end.B. Radial Vipp1–GFP fluorescence (green) and chlorophyll fluorescence (red) after 0, 10, 20, 30, 40 min HL exposure, combining data from two replicates on a standardised radial scale.C. Regional density of puncta over duration of HL exposure with S.E.M., showing puncta density for cytoplasm (blue), thylakoid (green) and near the plasma membrane (red). WT (LL) is shown at the end. Data based on two images per time point with 7–79 cells per image.D. Confocal fluorescence micrographs showing the distribution of Vipp1–GFP fluorescence after 5, 15, 30, 60 min of HL exposure as indicated. Chlorophyll fluorescence is shown in red and GFP fluorescence in green. Scale bar: 5 microns.

Under LL growth (8 μE m^−2^ s^−1^), the distribution of GFP fluorescence in *Synechocystis vipp1–gfp* was diffuse (Fig. 1A–D). The distribution of the majority of GFP fluorescence under these conditions resembles the distribution of free GFP or hydrophilic dyes (Fig. 2A) which show strong fluorescence in the central cytoplasm, and weaker fluorescence towards the periphery of the cell where the cytoplasm is ‘diluted’ by the thylakoid membranes (Mullineaux *et al*., 2008); see Fig. S4 for the distribution of free GFP in *Synechocystis*. While the majority of Vipp1–GFP under LL is dispersed in the cytoplasm, there are also some bright fluorescent spots indicating concentrations of Vipp1–GFP at the outer periphery of the thylakoid membranes (Figs 1A–D and 2A). Exposure to HL (600 μE m^−2^ s^−1^) led to dramatic changes in the distribution of Vipp1–GFP (Fig. 1E–H), with a shift from the cytosol towards the cell periphery (Fig. 2A), combined with coalescence of Vipp1 into puncta resulting in a large increase in the number of puncta per cell (Fig. 2B). No such effects were seen with free GFP, which showed no significant puncta counts under any condition (Fig. S4). This confirms that protein coalescence reflects the behaviour of Vipp1 rather than the GFP tag. The majority of Vipp1–GFP puncta were found in the vicinity of the plasma and thylakoid membranes (Fig. 2C). At our optical resolution of 200–300 nm we cannot be sure whether these puncta are directly associated with membrane surfaces, or merely in the same region of the cell as that occupied by the membranes. A few puncta were found in the central cytoplasm and are therefore presumably not directly membrane-associated (Figs 1E–H and 2A). The number of puncta near the plasma membrane was similar under LL and HL, but HL exposure resulted in a massive increase in the number of puncta near the thylakoids (Fig. 2A and C). Puncta formation and redistribution of Vipp1–GFP were detectable after 5 min of HL exposure and complete after about 50 min (Fig. 3). GFP fluorescence intensity indicates distinct populations of brighter and dimmer puncta (Fig. S5); distinct localisation of the brighter and dimmer puncta could be detected after about 35 min, with the dimmer puncta located closer to the centre of the cell, while the brighter puncta remained distal (Fig. S5B).

The GFP content of puncta was estimated by comparison with the fluorescence intensity of previously characterised puncta in *Escherichia coli,* on the assumption that fluorescence yields per GFP are similar (Lenn *et al*., 2008; Liu *et al*., 2012). The brightest *Synechocystis* puncta contained 280 ± 20 Vipp1–GFP, whereas the dimmer ones contained 130 ± 20 Vipp1–GFP (Fig. S6). The mean diameter of the puncta (corrected by deconvolution with the point-spread function of the microscope) was estimated as 100 ± 25 nm (± SD, *n* = 60).

### Proteins associated with Vipp1 puncta in *Synechocystis*

**Figure 4:**
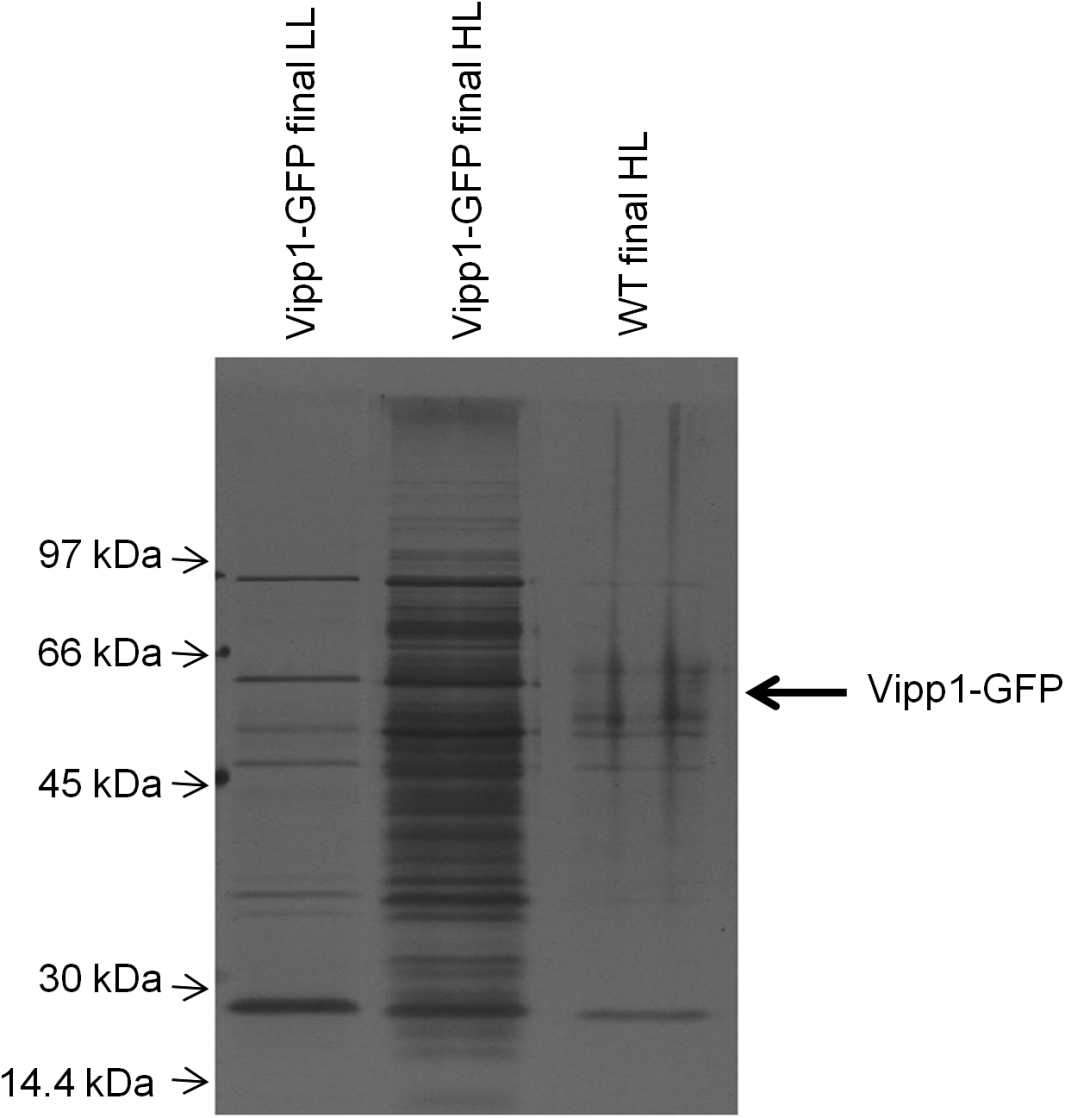
Isolation of Vipp1–GFP-associated proteins in *Synechocystis* by anti-GFP affinity binding. Silver-stained gel showing the final GFP affinity-bound fractions from *Synechocystis* *vipp1–gfp* LL and HL, and wild-type HL. Samples contained material extracted from equal numbers of cells (as judged from turbidity).

To examine the composition of the *Synechocystis* Vipp1–GFP puncta, we used pull-downs with magnetic beads functionalised with anti-GFP antibody, with cell disruption carried out in the absence of detergent. SDS-PAGE of the isolated Vipp1–GFP-associated fraction from HL-exposed *vipp1–gfp* cells consistently showed numerous protein bands absent (or present only below detection thresholds) in preparations from HL-exposed WT cells and from LL-grown *vipp1–gfp* cells (Fig. 4). Some protein bands were present regardless of strain and conditions and therefore represent background contamination. However, many bands were present exclusively in *vipp1–gfp* cells exposed to HL, suggesting that these proteins are directly or indirectly associated with the Vipp1–GFP puncta formed under these conditions (Fig. 4). In combination with the fluorescence imaging (Figs 1 and 3) this suggests that Vipp1 is mainly dispersed, or assembled only as homo-oligomers (Fuhrmann *et al*., 2009a) in the cytoplasm under LL, but HL exposure triggers the recruitment of Vipp1 into assemblies with numerous associated proteins.

**Figure 5:**
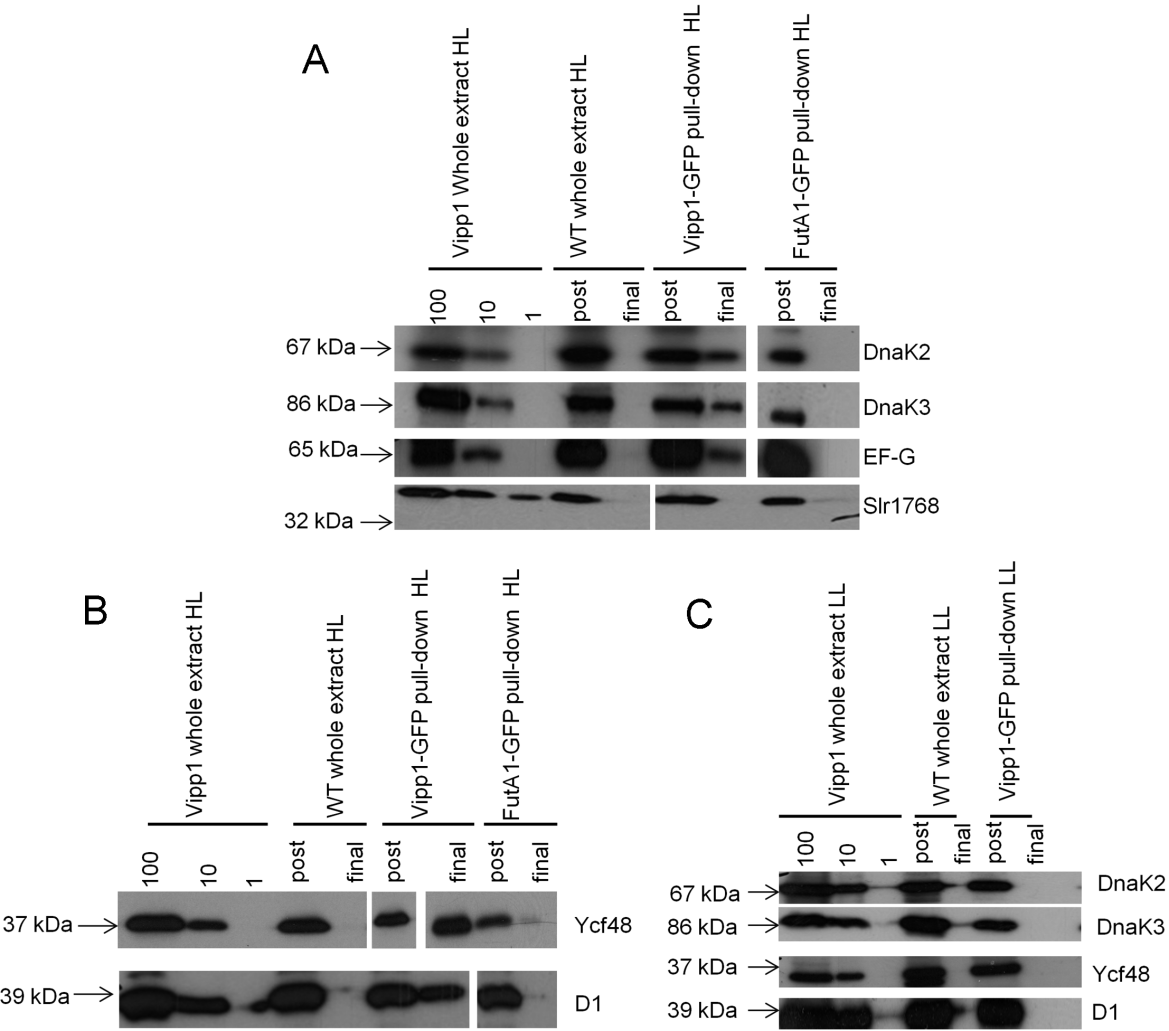
Verification by immunoblotting of Vipp1 interaction partners in *Synechocystis* identified by mass spectrometry.A. Immunoblots on fractions isolated from HL-treated wild-type, *vipp1–gfp* and *futA1–gfp* cells, showing that DnaK2, DnaK3 and EF-G (but not Slr1768) are retained in the GFP affinity-bound fraction specifically in *vipp1–gfp* cells. Spaces shown between the lanes on the blots for DnaK3, EF-G and Slr1768 indicate combination of data from different places on the blot, but all data are from the same blots. Sample dilutions (100%, 10%, and 1%) for unfractionated (whole extract) *Synechocystis vipp1–gfp* are shown for comparison. The first (post) elution is the fraction washed through the column, whereas the final elution is the fraction retained by GFP-affinity binding.B. Immunoblots showing that the Ycf48 and D1 proteins are affinity-bound in HL *vipp1–gfp* cells, but not wild-type or *futA1–gfp.* Spaces shown between the lanes on the blots for D1 and Ycf48 indicate combination of data from different places on the blot, but all data are from the same blots.C. DnaK2, DnaK3, Ycf48 and D1 are not affinity-bound in LL *vipp1–gfp* cells (in contrast to HL *vipp1–gfp* cells, see A, B).

Bands from the *Synechocystis* Vipp1–GFP associated fraction were identified by mass spectrometry (Table 1, Fig. S7, Table S1). A significant number of proteins could be detected above threshold only in *vipp1–gfp* cells and specifically after HL exposure. Such proteins include components of the photosynthetic apparatus (D1 from Photosystem II, PsaA from Photosystem I and phycobilisome subunits). In addition, a number of the proteins detected are implicated in stress responses, including the orange carotenoid-binding protein (OCP) (Kirilovsky and Kerfeld, 2012), the product of ORF *sll0755*, which is a stress-induced thioredoxin-dependent peroxiredoxin whose expression increases during HL exposure (Pérez-Pérez *et al*., 2009) and LrtA, a regulatory protein whose expression is increased under salt stress (Huang *et al*., 2006). There were numerous proteins implicated in polypeptide synthesis and various stages of protein assembly and maturation, including Elongation Factor G1 (EF-G, whose activity is regulated by photosynthetic electron transport in *Synechocystis* (Kojima *et al*., 2009), a tRNA amidotransferase and a tRNA ligase, the light-dependent protochlorophyllide reductase (whose activity is intimately linked to the biogenesis of photosynthetic complexes (Schottkowski *et al*., 2009) and the chaperone proteins GroL, DnaK2 and DnaK3. DnaK2 is important in stress responses in *Synechocystis* (Rupprecht *et al*., 2007; 2010[Bibr b39],[Bibr b40]), while DnaK3 has been suggested to be involved in protein targeting to thylakoids (Rupprecht *et al*., 2007; 2010[Bibr b39],[Bibr b40]). To verify these findings, selected proteins from different functional categories (Table 1) were examined semi-quantitatively by immunoblotting, comparing fractions isolated from HL and LL cells, and comparing *Synechocystis vipp1–gfp* with wild-type and a strain in which another protein, FutA1 (Katoh *et al*., 2001), was GFP-tagged (Fig. 5). Dilutions of unfractionated cell extracts from *Synechocystis vipp1–gfp* were used for comparison (Fig. S2 and Fig. 5). As expected, Vipp1–GFP was affinity-bound in both HL and LL *vipp1–gfp* cells, with nothing detected in wild-type cells (Fig. S2A). DnaK2, DnaK3 and EF-G were associated with the GFP affinity-bound fraction in HL *vipp1–gfp*, but not in wild-type or *futA1–gfp* cells (Fig. 5A). Immunoblotting did not detect the Slr1768 protein (Bryan *et al*., 2011) in any of the Vipp1–GFP associated fractions (Fig. 5A), confirming that only a specific subset of proteins associate with Vipp1 in HL conditions. Immunoblotting additionally showed the association of both the PSII assembly/repair factor Ycf48 (Komenda *et al*., 2008) and the D1 protein of PS II with Vipp1–GFP in HL cells (Fig. 5B). D1 was not detected by mass spectrometry in *Synechocystis* (Table 1, Table S1) while mass spectrometry gave an indication of Ycf48 protein but with a signal too low for accurate sequence determination. DnaK2, DnaK3, Ycf48 and D1 could not be detected in the GFP-affinity bound fraction from LL cells (Fig. 5C), although Vipp1–GFP itself is still bound (Fig. S2B). This confirms that the association of these proteins with Vipp1 occurs specifically after HL exposure.

**Table 1:** Proteins co-purifying with Vipp1–GFP in HL-treated *Synechocystis* cells

Predicted functional group	Protein identified by mass spectrometry from Vipp1–GFP-associated fraction from HL-treated *Synechocystis* 6803
Translation and RNA processing	Asn/Gln-tRNA amidotransferase subunit B (*gatB*) Elongation factor G1 (*fusA*) Elongation factor Tu (*tuf*)^a^ Glycine-tRNA ligase beta subunit (*glyS*) GTP-binding protein TypA/BipA homologue (*typA*) Polyribonucleotide nucleotidyltransferase (*pnp*) Ribonuclease II (*rnb*) Translation initiation factor IF-2 (*infB*)
Assembly and translocation of protein complexes	Chaperone protein DnaK2 (*dnaK2)* Chaperone protein DnaK3 (*dnaK3)* Chaperonin 1 (*groL1*) Light-dependent protochlorophyllide reductase (*por*) Protein translocase subunit SecA (*secA*)
Photosynthesis, electron transport or oxidative phosphorylation	Allophycocyanin alpha chain (*apcA*) ATP synthase subunit beta (*atpD*) C-phycocyanin alpha chain (*cpcA*) C-phycocyanin beta chain (*cpcB*) Ferredoxin-NADP reductase (*petH*) Photosystem I protein A1 (*psaA*) Phycobiliprotein ApcE (*apcE*)^a^ Phycobilisome linker polypeptide (*cpcC1*)^a^ Phycobilisome linker polypeptide (*cpcC2*)^a^ Ribulose bisphosphate carboxylase large chain (*cbbL*)
Stress-related proteins	Light repressed protein A homologue (*lrtA*) Peroxiredoxin (*sll1621*) Orange carotenoid-binding protein (*ocp*) Polyphosphate kinase (*ppk*) Thiol-specific antioxidant protein (*sll0755*) Phytoene dehydrogenase (*pds*)
Miscellaneous	Aconitate hydratase 2 (*acnB*) Argininosuccinate synthase (*argG*) Green fluorescent protein (*gfp*)^a^ Phosphoglycerate kinase (*pgk*) Phosphoribulokinase (*prk*) Phototaxis receptor PixJ1 (*pixJ1*) Sugar kinase slr0537 (*slr0537*) Transketolase (*tktA*) Vipp1 (*vipp1*)^a^ (P)ppGpp 3′-pyrophosphohydrolase (*spoT*)

a Also detected in LL-adapted cells, therefore not specifically associated with Vipp1–GFP under HL.

Proteins were identified by mass spectrometry, and were detected only after HL treatment unless otherwise indicated. Full details in Table S1.

**Figure 6:**
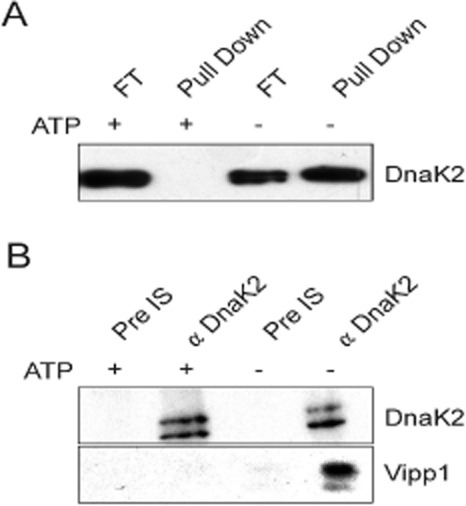
Specificity of the DnaK2–Vipp1 interaction tested by pull-down and co-immune precipitation experiments.A. Vipp1 was heterologously expressed in *E. coli* as a fusion protein containing an N-terminal His-tag and purified as described in detail in Fuhrmann *et al*. (2009b). The His-tagged protein was not eluted from the Ni-NTA-matrix but incubated with *Synechocystis* cellular extract in the presence of 5 mM ATP (+ ATP) or 10 U apyrase (− ATP). Binding of DnaK2 to Vipp1 was subsequently analysed by SDS-PAGE and Western blotting using an anti-DnaK2 antibody (Rupprecht *et al*., 2007).B. Anti-DnaK2 antiserum as well as pre-immune serum was coupled to protein A sepharose (Sigma) and incubated with *Synechocystis* cellular extracts. After rigorous washing co-precipitated proteins were analysed by SDS-PAGE and Western blotting using antibodies directed against DnaK2 and Vipp1.

To verify the interactions with Vipp1 detected by our anti-GFP pull-down procedure, interaction between Vipp1 and a selected protein (DnaK2) was confirmed independently by expressing Vipp1 with an N-terminal His-tag in *E. coli* and immobilising the tagged protein on an Ni-NTA matrix. The immobilised Vipp1 protein bound DnaK2 from a *Synechocystis* cell extract (Fig. 6A). Co-immune precipitation from *Synechocystis* cell extracts with both Vipp1 and DnaK2 antibodies showed that Vipp1 interacts with DnaK2 and *vice versa* (Fig. 6B). Note that these results were from cells which had not been exposed to HL, therefore they indicate some Vipp1–DnaK2 interaction even in LL cells. The extent of this interaction must presumably be below our detection thresholds in the anti-GFP pull-downs (Fig. 5).

### Spatial distribution of proteins associated with Vipp1 puncta

**Figure 7:**
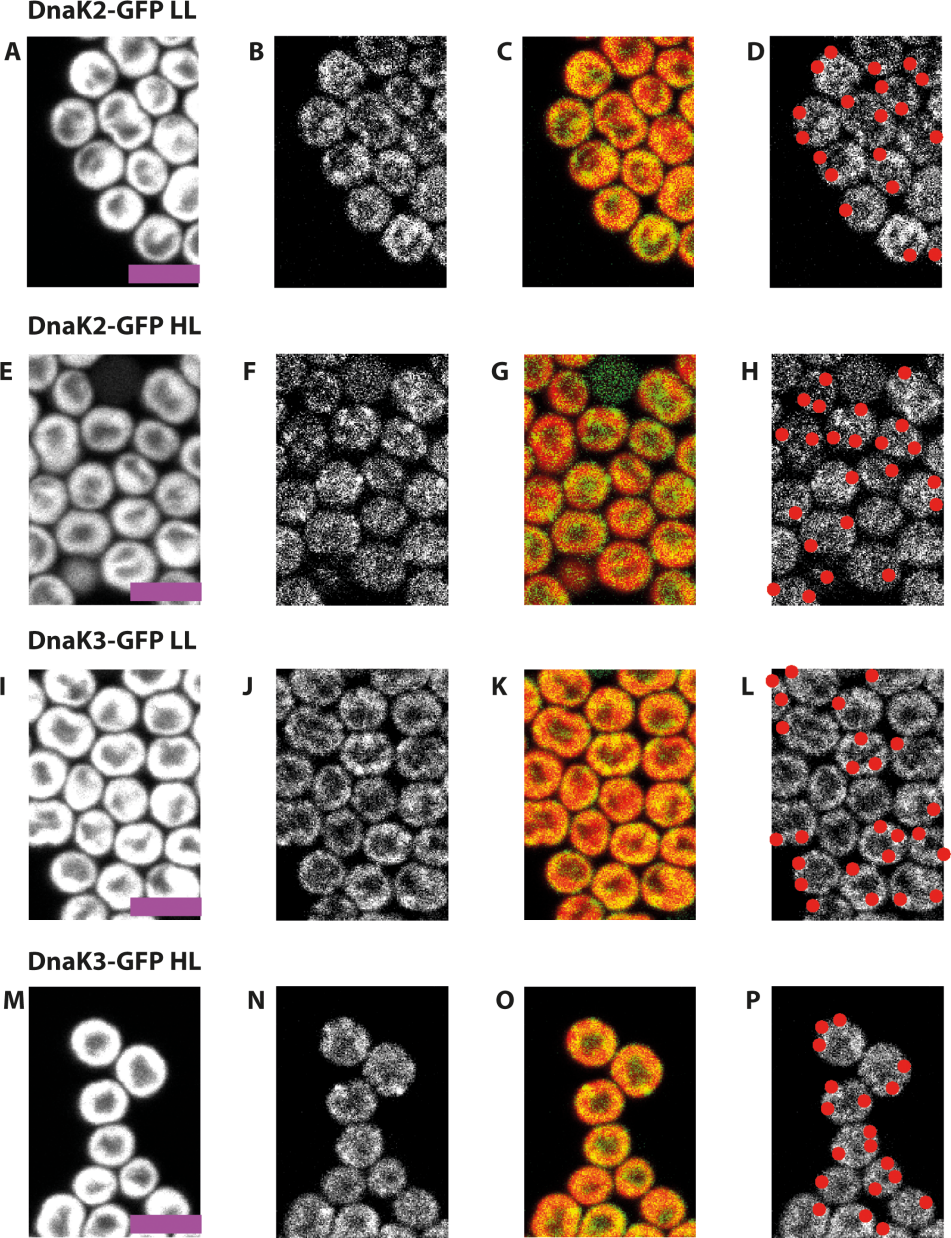
DnaK2/3 localisation in *Synechocystis*. Confocal fluorescence micrographs showing chlorophyll fluorescence (first column), GFP fluorescence (second column), chlorophyll (red):GFP (green) overlay (third column) and located puncta in red (fourth column). *DnaK2–gfp* cells under low light (LL) (A–D) and after high light (HL) exposure for 30 min (E–H). *DnaK3–gfp* cells under low light (LL) (I–L) and after high light (HL) exposure for 30 min (M–P). Scale bar: 5 microns.

**Figure 8:**
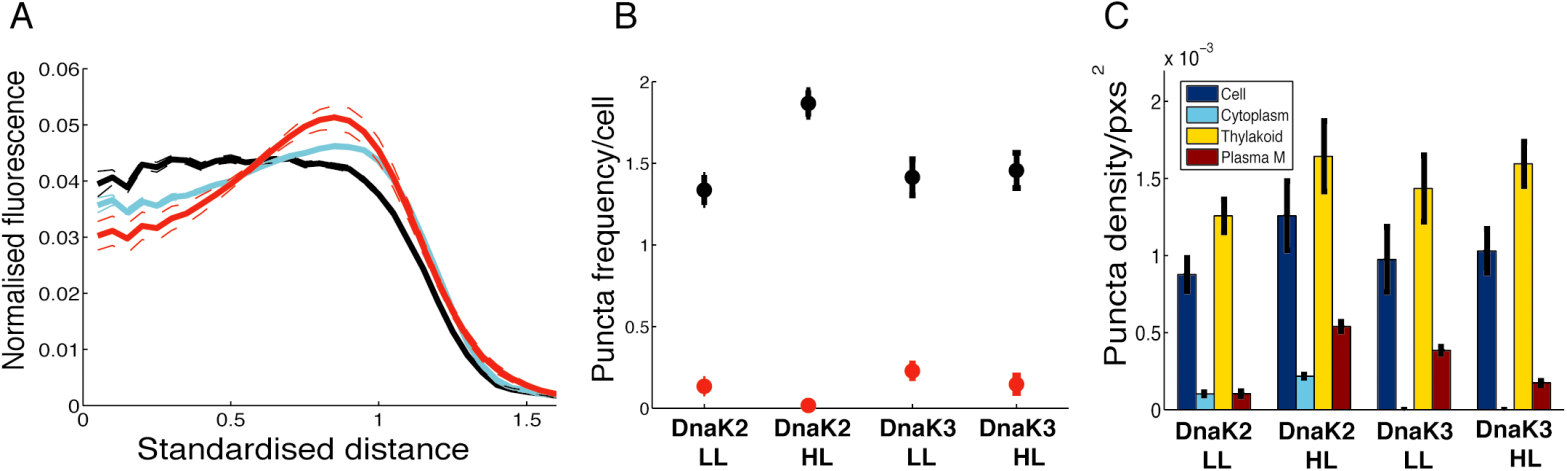
DnaK2/3 radial distribution and patterning in *Synechocystis*.A. Relative radial distribution (standardised radius) of DnaK2/3-GFP fluorescence under HL (black – DnaK2; cyan – DnaK3). Dashed lines are S.E.M. Chlorophyll shown in red, with S.E.M. Standardised distance refers to rescaling the ½ maximum radius for chlorophyll to a radial distance of 1.B. Average counts of puncta per cell in LL, HL for DnaK2/3. Red points are data after bleaching to reduce GFP fluorescence, with S.E.M.C. Density of puncta (in cell, cytoplasm, thylakoid, near to plasma membrane). Error bars show S.E.M. Data are from 125–230 cells depending on condition.

Proteins associated with Vipp1 in puncta under HL conditions would be expected also to show a punctate distribution, although the strength of any spatial heterogeneity and localisation would depend on the proportion of the population associated with Vipp1. We used GFP-tagging and fluorescence microscopy to examine the localisation of two of the Vipp1 interaction partners identified from the pull-down assays, DnaK2 and DnaK3. We created C-terminal GFP fusions for both DnaK2 and DnaK3. Both gene fusions were expressed from the native chromosomal loci of *Synechocystis* and PCR was used to confirm that the transformants were fully segregated (Fig. S8). As both genes are essential in *Synechocystis* (Rupprecht *et al*., 2007), complete segregation of the mutant strains and replacement of the wild-type by the fusion genes demonstrates that the fusion proteins were functional. We found that both DnaK2/3 had average radial distributions consistent with thylakoid association (Figs 7 and 8), with a greater proportion of DnaK3 appearing thylakoid-associated (Fig. 8A). Both proteins formed puncta (Fig. 7), predominantly in the thylakoid region (Fig. 8C). Puncta formation was weaker than for Vipp1, although GFP signals were lower in these cases making quantification more difficult because of image noise. DnaK2 showed a significant increase in puncta counts under HL relative to LL (Fig. 8) but DnaK3 puncta counts remained invariant. The punctate distribution of DnaK2 and DnaK3 in the thylakoids (Figs 7 and 8) is similar to the distribution of Vipp1 in HL-treated cells (Figs 1 and 2), consistent with the interactions that we detected by pull-downs and co-immune precipitation (Figs 5 and 6; Table 1). The HL-induced increase in the DnaK2 puncta count is less strong than for Vipp1, and there is no significant HL-induced increase in the DnaK3 puncta count. This suggests that there is increased colocalisation of DnaK2/3 and Vipp1 under HL, which is again fully consistent with our biochemical data. A simple interpretation would be that HL treatment induces the binding of Vipp1 to pre-existing localised bodies in the thylakoid region containing DnaK2 and DnaK3.

### Spatial distribution and relocalisation of Vipp1 in *Synechococcus*

**Figure 9:**
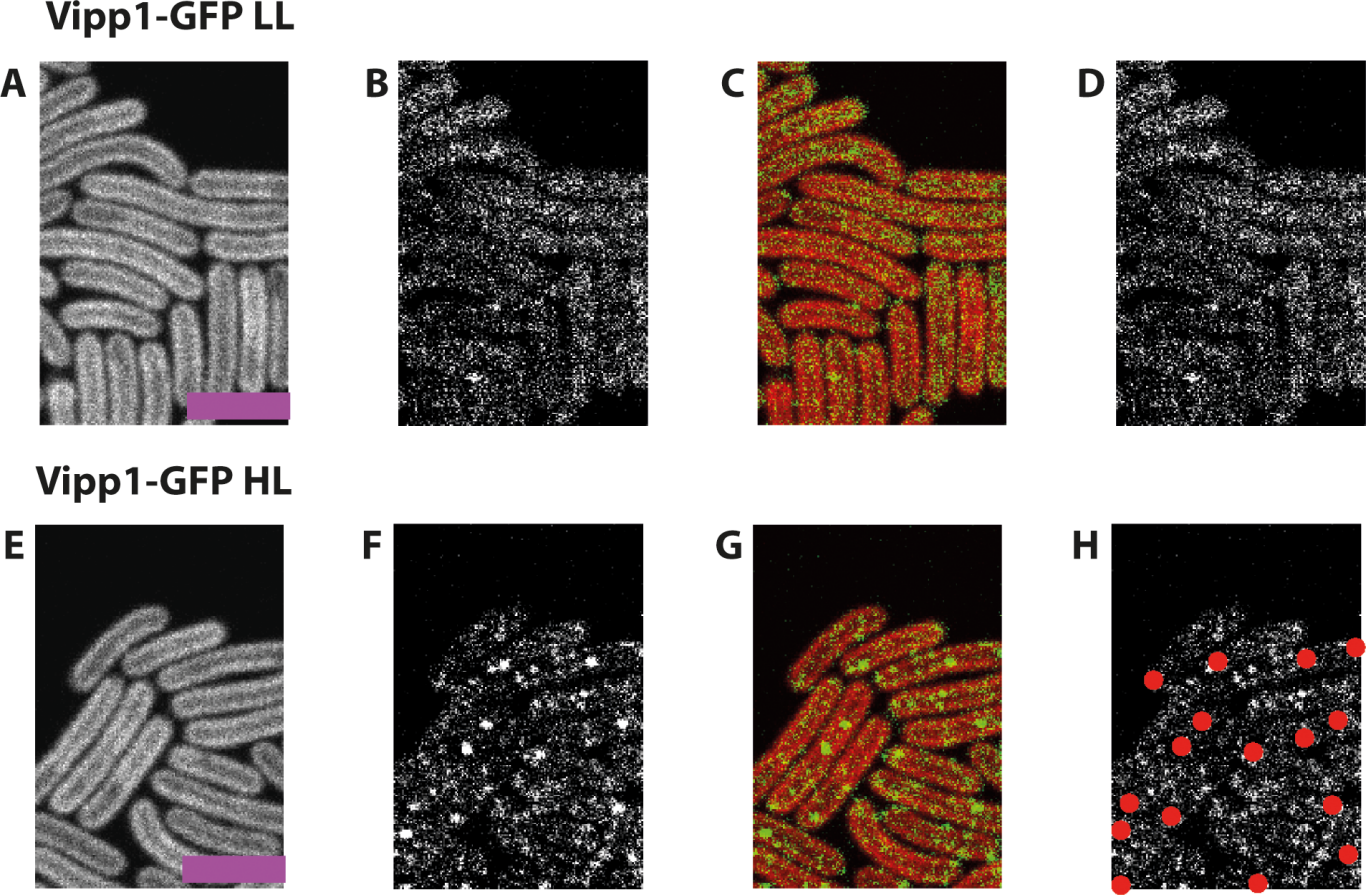
Vipp1 redistributes under high-light in *Synechococcus.* Confocal fluorescence micrographs showing chlorophyll fluorescence (first column), GFP fluorescence (second column), chlorophyll (red):GFP (green) overlay (third column) and located puncta in red (fourth column). *Vipp1–gfp* cells under LL (A–D), and after exposure to HL for 30 min (E–H). Scale bar: 5 microns.

**Figure 10:**
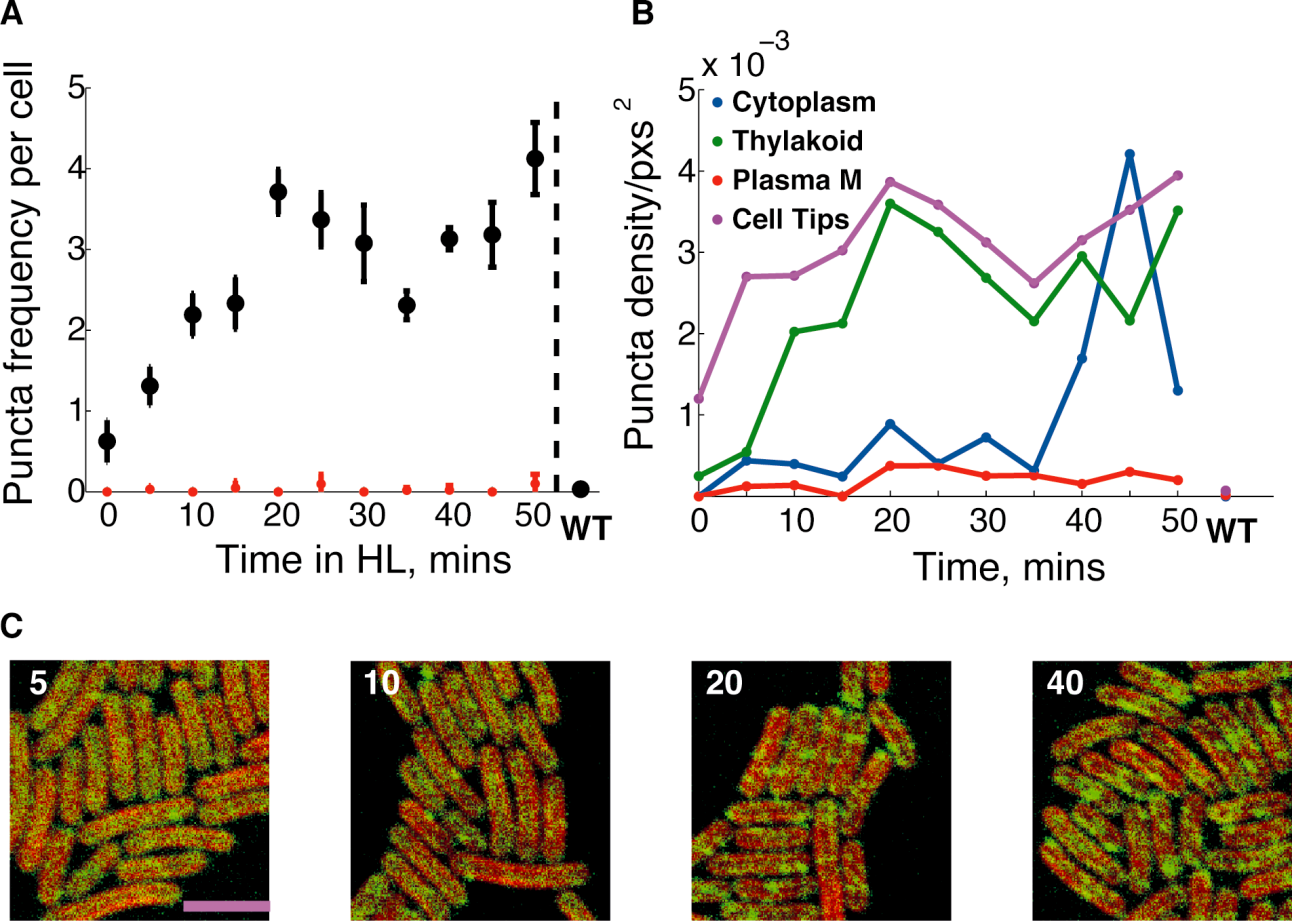
Vipp1 distribution and patterning in *Synechococcus.*A. Average puncta per cell counts under HL exposure for stated time, after bleaching in red, with S.E.M. WT (HL) is shown at the end. Cells originally LL adapted (0 min)B. Density of puncta by region (cytoplasm, thylakoid, near plasma membrane, and cell tips). Data from on 124–165 cells over a time-course with 1 image per time point.C. Confocal fluorescence micrographs showing puncta formation after 5, 10, 20, 40 min (indicated top left) of HL exposure after adaptation to LL, chlorophyll (red):GFP (green) overlay. Scale bar: 5 microns.

We extended our study by examining the spatial localisation of Vipp1–GFP in a second species of cyanobacterium, *Synechococcus* sp. PCC7942*. Synechococcus vipp1–gfp* cells were fully segregated (Fig. S1B) and only slightly more light-sensitive than the wild-type (Fig. S3F), suggesting little perturbation of Vipp1 function. Vipp1–GFP in *Synechococcus* exhibited similar localisation and dynamics to *Synechocystis* with formation of puncta under HL (Fig. 9). Specifically, under LL growth (8 μE m^−2^ s^−1^), the distribution of GFP fluorescence was diffuse in the cytoplasm (Fig. 9A–D), while puncta formation was induced by HL exposure (600 μE m^−2^ s^−1^) (Fig. 9E–H), being typically faster than in *Synechocystis* with puncta appearing within 10 min (Fig. 10). Puncta initially appeared close to the poles of the cell (Fig. 10B). Again, there were populations of brighter and dimmer puncta, but with generally lower Vipp1–GFP content than in *Synechocystis* (Fig. S6C). Thus, HL relocalisation and aggregation is probably ubiquitous among the cyanobacteria.

### Stability and mobility of Vipp1–GFP puncta

**Figure 11:**
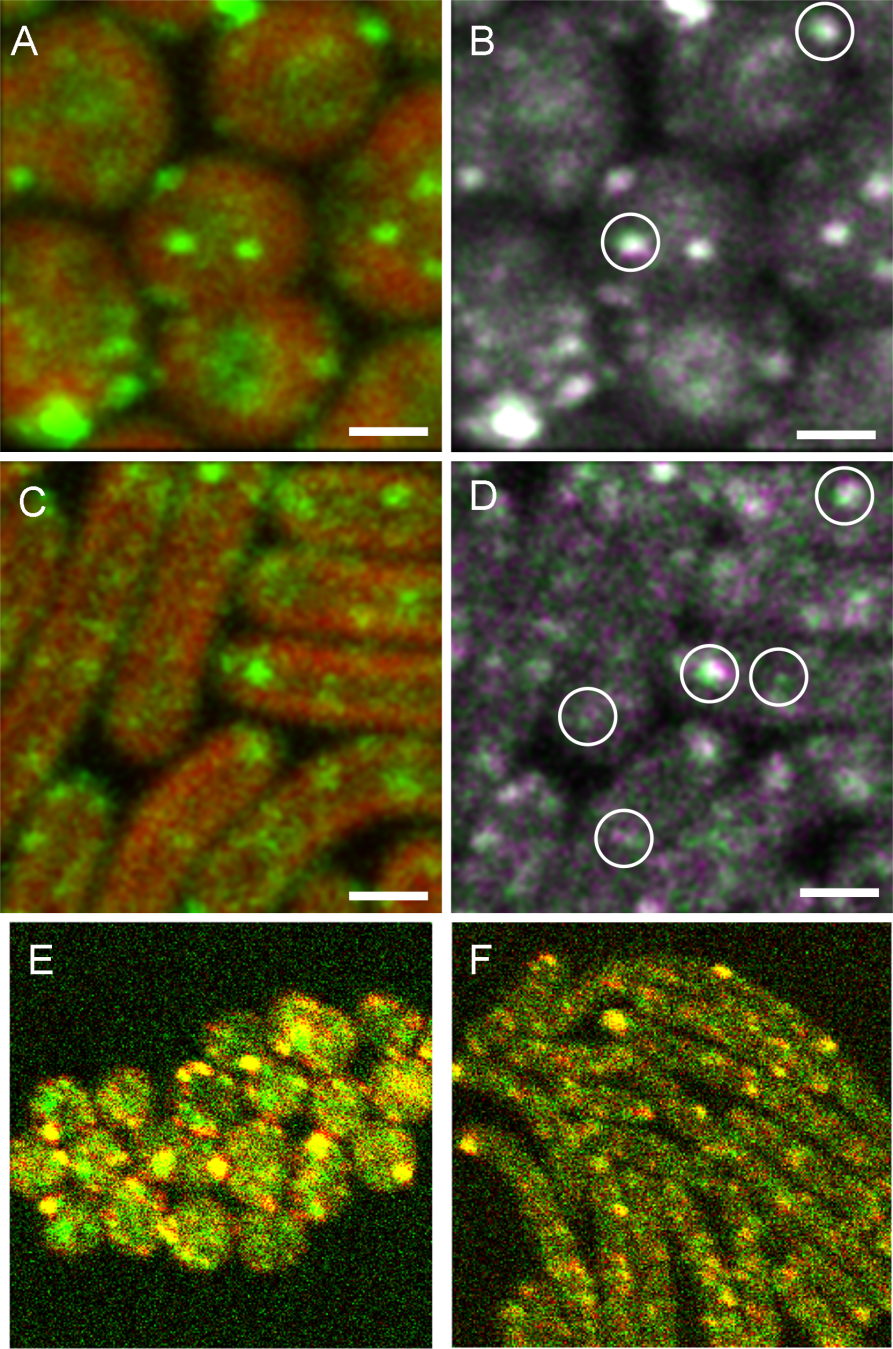
Stability and mobility of Vipp1–GFP puncta in *Synechocystis* (A, B, E) and *Synechococcus* (C, D, F). Cells were exposed to HL for 1 h to induce puncta formation.A and C. Frames taken from time-lapse movies (see Supporting Movies S1 and S2) with overlay of GFP fluorescence (green) and chlorophyll fluorescence (red).B and D. Overlaid GFP fluorescence images from successive frames. The first frame is the same as in (A) and (C) and shown in green: the second frame is recorded 200 ms later and shown in magenta. Circles highlight examples of puncta whose position changes between the frames. Scale bars: 1 μm.E and F. Red/Green overlay of Vipp1–GFP fluorescence images recorded with a 30 min interval.

We used time-lapse image sequences to examine the movement of Vipp1–GFP puncta in *Synechocystis* and *Synechococcus*. Some, but not all, of the HL-induced puncta showed detectable but very confined movement on a timescale of seconds (Fig. 11; Supporting Movies S1 and S2). We applied nanoscale single-particle tracking to representative mobile puncta in *Synechococcus* for up to a few seconds duration on individual molecular trajectories (longer continuous tracking was limited by bleaching of the GFP). Diffusional analysis (Xue and Leake, 2009; Xue *et al*., 2010; Robson *et al*., 2013) indicated predominantly isotropic Brownian diffusion on these short timescales (Fig. S9). To examine the longer-term stability of puncta, we also recorded time-lapse images at 5 min intervals over a 30 min period. This demonstrates that the majority of bright puncta are long-lived and immobile, or mobile only within very confined domains, for timescales of 30 min or greater (Fig. 11).

## Discussion

Our results show that rapid and dramatic redistribution of Vipp1 occurs in cyanobacterial cells under HL (Figs 11, 2, 9 and 10). Stress triggers, by an unknown mechanism, the recruitment of Vipp1 from a predominantly dispersed pre-existing cytosolic pool into small, localised puncta around 100 nm in diameter and containing around 100–300 Vipp1 molecules (Fig. S5). *Synechocystis* Vipp1 forms oligomeric rings with molecular masses exceeding 2 MDa and 25–33 nm in diameter (Fuhrmann *et al*., 2009b). The smallest puncta that we observe *in vivo* could correspond to 1–2 of these rings, and the largest to about 5–6. Although our data provide no information on the structural organisation of Vipp1 in the puncta, it is possible that the larger assemblages result from the stacking of rings into rod structures, as occasionally observed for *Synechocystis* Vipp1 (Fuhrmann *et al*., 2009b). Punctate distribution of Vipp1 *in vivo* was previously observed in *Chlamydomonas* by immunofluorescence microscopy (Nordhues *et al*., 2012), while GFP-tagging and confocal microscopy revealed a variety of dynamic structures in *Arabidopsis* chloroplasts, including puncta, rods and lattice-like structures (Zhang *et al*., 2012). However, neither of these studies looked at the dynamics and the effects of HL treatment on Vipp1 distribution.

The HL-induced cyanobacterial Vipp1 puncta concentrate in the vicinity of the thylakoid and cytoplasmic membranes (Figs 11 and 2), and near the poles in the rod-shaped cells of *Synechococcus* (Figs 9 and 10)*.* We found that the same HL treatment led to the formation of biochemically detectable direct or indirect interactions of Vipp1 with an extensive collection of proteins. Previously identified proteins interacting with Vipp1 in chloroplasts include the chaperonins CDJ2, GrpE, HSP70B, HSP90C and HSP90.5 (Liu *et al*., 2005; Heide *et al*., 2009; Feng *et al*., 2014) and the membrane insertion/photosystem biogenesis factor Alb3.2 (Göhre *et al*., 2006). In the present study we could detect few interaction partners of the *Synechocystis* Vipp1 under LL, but, following HL treatment, a diverse collection of proteins co-isolate with Vipp1, including photosynthetic components, proteins associated with stress responses and components implicated in all stages of the synthesis and maturation of protein complexes (Table 1). The recruitment of these interaction partners correlates with puncta formation, suggesting that these proteins are present either directly in the Vipp1 puncta, or in membrane fragments or other bodies linked to the puncta.

We used GFP-tagging and fluorescence microscopy to examine the subcellular distribution of two biochemically detected Vipp1 interaction partners, the chaperones DnaK2 and DnaK3 (Figs 7 and 8). Both DnaK2 and DnaK3 proved to be concentrated in the thylakoid membrane region and formed puncta. However, in contrast to Vipp1, puncta formation was not greatly influenced by HL exposure, with the only effect being a slight increase in DnaK2 puncta count (Fig. 8). Thus, the localisation of DnaK2 and DnaK3 appears similar to Vipp1 in HL, but distinct in LL. This is entirely consistent with our biochemical approach, which indicates increased *in vivo* association of Vipp1 with DnaK2 and DnaK3 after HL exposure (Table 1, Fig. 5). The simplest interpretation of the data would be that HL exposure triggers the association of Vipp1 with pre-existing entities of membrane-bound DnaK2 and DnaK3.

The redistribution of Vipp1 under HL suggests a specific role for Vipp1 in light-stress responses. This is consistent with the stronger requirement of Vipp1 for thylakoid membrane maintenance under HL stress in *Chlamydomonas* chloroplasts (Nordhues *et al*., 2012), while the upregulation of Vipp1 synthesis under salt stress (Huang *et al*., 2006) suggests that Vipp1 may also play a role in the response to other stress conditions. The HL induced aggregation of Vipp1 into puncta may reflect the switch from a resting to an active state. A number of other related proteins show similar behaviour. PspA, a protein related to Vipp1, is not confined to cyanobacteria, but widespread in prokaryotes (Westphal *et al*., 2001; Vothknecht *et al*., 2012). In *E. coli*, PspA is important for maintaining membrane integrity under stress conditions and under such conditions, PspA is concentrated in localised, mobile puncta close to the cytoplasmic membrane (Engl *et al*., 2009). PspA also shows striking changes in its behaviour and subcellular distribution in *Yersinia enterocolitica* under stress conditions (Yamaguchi *et al*., 2013). Under non-inducing conditions all four Psp proteins appear as highly mobile foci either in the cytoplasm or at the cytoplasmic membrane. However upon exposure to stress they form large static complexes at the cytoplasmic membrane (Yamaguchi *et al*., 2013). In *Bacillus subtilis* the LiaI protein is organised into a few membrane-anchored foci; following stress the LiaI foci recruit the PspA homologue LiaH to the membrane and the foci subsequently become mostly static (Domínguez-Escobar *et al*., 2014). Vipp1 foci also show dynamic movement (Zhang *et al*., 2012); however, unlike both PspA and LiaH this movement only occurs under stress conditions. Therefore, stress-induced re-localisation may be important to the mode of action of PspA/IM30 proteins, which function through larger mobile or static complexes through protein–protein interactions at membrane surfaces (Domínguez-Escobar *et al*., 2014).

Since Vipp1 was suggested to be involved in the formation of vesicles transferring material from the cytoplasmic membrane to the thylakoids (Kroll *et al*., 2001; Westphal *et al*., 2001), we considered the possibility that the Vipp1 puncta are transport vesicles. However, their behaviour does not appear consistent with this idea. Some of the puncta are mobile (Fig. 11 and Fig. S9), but their movement is limited to confined regions of the cytoplasm (Fig. 11). Furthermore, the puncta appear too long-lived to be transient transport vesicles, the brightest ones being stable for at least 30 min (Fig. 11). The presence of polypeptide synthesis and assembly factors from Elongation Factor G1 to the chaperonins GroL, DnaK2 and DnaK3 (Table 1) suggests that the puncta could have a direct role in protein assembly, or are linked to protein assembly zones in the membrane, or are in close proximity to such assembly zones.

The assembly of photosynthetic complexes in *Synechocystis* has been reported to be localised in defined biogenesis centres at the cell periphery (Stengel *et al*., 2012), identified as an isolated membrane fraction defined by the presence of the PratA PSII assembly factor (Rengstl *et al*., 2011). The location of some (but not all) of the Vipp1 puncta that we observe is consistent with the location of these centres at the cell periphery (Fig. 11M), and would be consistent with the suggestion that Vipp1 is a dynamic structural component of these assembly centres (Rütgers and Schroda, 2013). Our results show association of the Vipp1 puncta with some of the protein components of the PratA-defined membrane, including the D1 protein of Photosystem II, the light-dependent protochlorophyllide reductase and the assembly factor Ycf48 (Komenda *et al*., 2008). However we could not detect other components of the PratA-defined membrane, notably PratA itself (Rengstl *et al*., 2011). Thus, the functional relationship of the PratA-defined membrane with the Vipp1 puncta remains to be determined.

As a working model, we suggest that the Vipp1-puncta may be part of centres that are important under stress conditions and are involved in rapid and efficient protein synthesis and/or complex assembly. The photosynthetic reaction centre subunits that we detected are among those whose rapid synthesis is required during HL stress, along with the energy-quenching OCP protein (Kirilovsky and Kerfeld, 2012) and the thioredoxin-dependent peroxiredoxin (Pérez-Pérez *et al*., 2009). This model would explain why Vipp1 content becomes critically important for the maintenance of thylakoid membranes under HL stress (Nordhues *et al*., 2012). The model is also consistent with the suggestion that Vipp1 is involved in delivering protein substrates to the thylakoid-localised Albino3.2 for membrane integration in *Chlamydomonas* chloroplasts (Göhre *et al*., 2006; Nordhues *et al*., 2012). The model is also fully consistent with the recently published work on the phenotype of the fully segregated *vipp1* null mutant of *Synechococcus* sp. PCC7002, which suggests a specific role for Vipp1 in delivering PSI polypeptides for insertion into the thylakoid membrane (Zhang *et al*., 2014). However, the structural basis for Vipp1 activity and the signalling pathway that triggers it, remain to be determined. Further experimental evidence will be required to establish definitively the relationship between Vipp1 bodies and sites of protein synthesis.

## Experimental procedures

### Bacterial strains and media

*Synechocystis* sp. PCC6803 (WT) (not the glucose tolerant strain) and *Synechococcus* sp. PCC7942 were grown photoautrophically in BG-11 medium (Castenholz, 1988) at 30°C under 8 μE m^−2^ s^−1^ white light in tissue culture flasks (Nunc), with continuous shaking. For HL, cells were either incubated in BG-11 at 30°C under 600 μE m^−2^ s^−1^ white light or spotted onto BG-11 plates and similarly illuminated. *E*. *coli* strains used were DH5α and BW25113 (*E. coli* stock centre).

### Transformation of cyanobacteria

*Synechocystis* sp. PCC6803 and *Synechococcus* sp. PCC7942 cells were transformed according to Chauvat *et al*. (1989). A culture in exponential growth was harvested and washed with fresh BG-11 and resuspended to 1 × 10^9^ cells ml^−1^. Approximately 10–50 μg of plasmid DNA was then added to 150 μl cell suspension and incubated at 50 μE m^−2^ s^−1^ white light at 30°C for 1–5 h before spreading onto BG-11 plates. Plates were incubated under 50 μE m^−2^ s^−1^ white light at 30°C for approximately 16 h. Increasing amounts of apramycin were then added and cells were further grown on selective plates containing a final concentration of 100 μg ml^−1^ apramycin.

### Generation of *Synechocystis* and *Synechococcus* *vipp1–gfp* strains

The *vipp1–GFP* strains were generated according to the REDIRECT manual (Gust *et al*., 2003), with minor modifications. The protocol and plasmids were provided by PBL Biomedical Laboratories. For both *Synechocystis* and *Synechococcus,* forward and reverse *vipp1* primers (6803 *sll0617*F-ccatcgggcagtaaatgacg and *sll0617*R-gtggcgcttacctagaaggg and 7942 *0794*F-agcgcggatcttgctgtaac and *0794*R-cagcatgggcaactgtcctg) were used to amplify a 3 kb region including *vipp1* flanked by 1 kb either side to assist with homologous recombination. The 3 kb PCR products were cloned into pGEM T-easy (Promega) as detailed in the Promega manual. *Vipp1–gfp* fusions were generated by amplifying the apramycin*–gfp* cassette from pIJ786 using two long PCR primers:

*sll0617*RF-gacgacctacgtcgtcggttaaataatctgtaattggttctgccgggcccggagctgcc
*sll0617*RR-taggttaatgggggatttttactggctgagttaaaccaaattccggggatccgtcgacc
*0794*RF-gcagaactagaagccctgaagcgcgaactcgacggactcctgccgggcccggagctgcc
*0794*RR-attgctgaagttgcagagtatgcagtttggaagaatgctattccggggatccgtcgacc

Each individual primer has at the 5′ end 39 nt matching either the *Synechocystis* or *Synechococcus* sequence either side of (but not including) the stop codon and a 3′ sequence (19 nt or 20 nt) matching the right or left end of the cassette. A full in-frame *gfp* fusion was generated via homologous recombination leading to the incorporation of *gfp* and a 21 nt linker region at the 3′ end of *vipp1*. Transformants were screened via PCR using the primers (6803FS-gccagtcaagatgatgcggtgatt and 6803RS-aaccgctgtcaggttacaggtcgt and 7942FS-gtggaagatgaactcgcagcaatg and 7942RS-ctgcttgccggagttgaattagtg) and sequenced using the T7 and S6 primer (Promega).

### Generation of *Synechocystis* *dnaK2 GFP* and *dnaK3 GFP* strains

The *dnaK2/3–GFP* strains were generated according to the REDIRECT manual as stated above. *Synechocystis* forward and reverse *dnaK2/3* primers; *sll0170*F-cgagacgatgttttccccagc and *sll0170*R-acaccgcacgaccaatgaaat and *sll1932*F-acgacaaaatccgccaacagg and *sll1932*R-ctacggtaaagctccaattcc were used to amplify a 3 kb region including *dnaK2/3* flanked by 1 kb either side to assist with homologous recombination. *DnaK2/3–gfp* fusions were generated by amplifying the apramycin*–gfp* cassette from pIJ786 using two long PCR primers:

*sll0170F*-gatgatgtcatcgatgcggaattctctgagccggagaaactgccgggcccggagctgcc
*sll0170R*-gggctaggggactttcaatacaggttttaaaacccagacattccggggatccgtcgacc
*sll1932F*-ttacaaaacggttgggatgatgacgatgatgattggttcctgccgggcccggagctgcc
*sll1932R*-caacggtttttaccctgtttggtcaacgaacttttgacaattccggggatccgtcgacc

Transformants were screened via PCR using the primers *dnaK2FS*-gtgtgaacgtgtcggccccag and *dnaK2RS*-aatccccctggttaaaggagg and *dnaK3FS*-gagcaaagaaaacaacatggg and *dnaK3RS*-ctcaggtcgaggttacagggg and sequenced using the T7 and S6 primer (Promega).

### Protein identification by mass spectrometry

Proteins were separated by 10% SDS-PAGE electrophoresis, stained and bands excised. Gel bands were treated with 25 mM NH_4_HCO_3_ in 25% acetonitrile (ACN) and washed twice with dH_2_O. Preparation of proteins for mass spectrometry was performed according to the manufacturer's protocol by in-gel digestion using OMX-S (OMX, Seefeld, Germany) (Granvogl *et al*., 2007). Peptide mixtures were desalted and concentrated on 20 μl StageTip C_18_-RP microcolumns (Thermo Fisher, Stockholm, Sweden) and eluted in 2–4 μl of 65% ACN, 1% 2-propanol, 0.1% formic acid (v/v). For electrospray ionisation, peptides were loaded into borosilicate nano ES emitters (Proxeon, Stockholm, Sweden), and sprayed at 0.8–1.5 kV (ESI +) and a cone voltage of 40 V in a nano-ESI source. MS and MS/MS analysis of peptides was performed using a Waters Q-Tof Premier mass spectrometer (Waters Corporation, Milford, MA, USA). MS spectra were recorded between 400 and 2000 *m/z* for at least 30 s (1 s/scan). MS/MS spectra were acquired using argon at collision energies between 26 and 40 eV. *De novo* sequence analysis (Plöscher *et al*., 2011) was performed using *MassLynx/Biolynx 4.2* software and the *b*- and *y*-ion series of spectra interpreted manually. Amino acid sequences were used for similarity search (http://www.ebi.ac.uk/Tools/fasta33) against the SwissProt- and TrEMBL- databases of the European bioinformatics institute (EBI) (http://www.expasy.org). Sequences were obtained from two independent SDS-PAGE separations. Sequence coverage (Supplementary Table S1) was calculated as the ratio of the number of amino acids in identified peptides divided by the number of amino acids in the complete protein sequence.

### Protein analysis and immunoblotting

Twenty millilitres of liquid cultures were grown to about 5 μM chlorophyll under LL (8 μE m^−2^ s^−1^ ) or exposed to HL (600 μE m^−2^ s^−1^ ). Cultures were normalised according to OD_750_ and cells were harvested, resuspended and washed twice in ACA buffer (750 mM ε-amino caproic acid, 50 mM BisTris/HCL pH = 7.0, 0.5 mM EDTA). The final volume of cell suspension was 500 μl to which 200 μl of glass beads (212 to 300 μm in diameter, Sigma-Aldrich, UK) were added. Cells were broken with a vortexer at 4°C using a 2 min on/2 min off cycle repeated four times. After cell breakage, 100 μl of each sample was retained as the pre-column sample. Fifty microlitres of Anti-GFP Microbeads (MACS Molecular) were added to the remaining lysate which was left on ice for 30 min. The μMACS column was placed in the magnetic field of the μMACS Separator, and prepared with 200 μl of μMACS lysis buffer (NaCl, 1% Triton, X-100, Tris HCl). The lysate was then added to the μMACS column as detailed for the μMACS Epitope Tag Protein Isolation kit (MACS Molecular). The flow-through was collected and the column was washed four times with 200 μl μMACS Wash Buffer 1, then once with 100 μl Wash Buffer 2. All fractions were collected. Twenty microlitres Elution Buffer (Tris HCl, DTT, 1% SDS, EDTA, Bromophenol blue, glycerol) preheated to 95°C was added onto the column and incubated for 5 min. Fifty microlitres preheated Elution Buffer was then added to elute the retained proteins. Twenty microlitres of each sample was loaded per lane onto a 10% (w/v) SDS-PAGE gel. Gels were either Coomassie-stained, silver-stained (Blum *et al*., 1987) or electro-blotted onto nitrocellulose membrane using the iBlot system (Invitrogen, UK) according to the manufacturer's instructions. Immunoblotting analysis was performed using specific primary antibodies and a horseradish peroxidase-conjugated secondary antibody (GE Healthcare, UK) with visualisation by a chemiluminescent kit (SuperSignal West Pico, Pierce, USA). Anti-GFP antibody was from Gentaur Molecular Products, Belgium. Other antibodies used were to prohibitin slr1768 (Boehm *et al*., 2009); Vipp1 (Fuhrmann *et al*., 2009a); DnaK2 (Rupprecht *et al*., 2007); D1 (residues 321–353 of precursor D1 from pea) (Nixon *et al*., 1990); Ycf48 (a rabbit polyclonal antiserum raised against *E. coli-*expressed *Synechocystis* Ycf48 with N-terminal His-tag).

### Fluorescence microscopy

Small blocks of BG11-agar with adsorbed cells on the surface were mounted in a custom-built sample holder with a glass coverslip pressed onto the cell layer. Laser-scanning confocal microscopy used a Leica TCS-SP5 with a 60× oil-immersion objective (NA 1.4) and 488 nm excitation from an Argon laser. Emission was recorded simultaneously at 502–512 nm for GFP and 670–720 nm for chlorophyll. The confocal pinhole was set to give *z-*resolution ∼ 0.8 μm for still images and ∼ 1.5–2 μm for dynamic image series (5 frames s^−1^).

### Regional fluorescence quantification

Chlorophyll fluorescence was used to demarcate cells and their regions (thylakoid, cytosol, cell periphery, and for *Synechococcus*, cell poles). Object extraction used a threshold that maximised the number of objects identified as cells (based on limits for cell area and cell eccentricity (*Synechocystis*) or cell width (*Synechococcus*) and a second (higher) threshold to define the inner cytosol region. GFP fluorescence was quantified in these regions. The bleached image was used to calibrate the autofluorescence, i.e. levels of GFP were defined relative to the bleached image. Radial fluorescence in *Synechocystis* and axial fluorescence in *Synechococcus* were determined on a per cell basis using chlorophyll fluorescence to define the cell geometry, i.e. radial (axial) coordinates were used to allow cell fluorescence to be averaged in each cell either radially (*Synechocystis*) or relative to the cell axis (*Synechococcus*). Averaging over cells was performed by using a linear rescaling to scale the ½ maximum radius for chlorophyll fluorescence to a radial distance of 1. Spots were determined by using a local fluorescence filter. Spot counts post-bleach and in wild-type were used to calibrate detection thresholds.

### Quantification of dimensions and GFP content of puncta

Yellow-green fluorescent microspheres (170 nm diameter; PS-Speck microscope point source kit, Invitrogen) were imaged using our standard microscope settings. Fluorescence profiles across the microsphere images were taken with ImageJ software (NIH Image, Bethesda, MD) and fitted to Gaussian curves. Similar fluorescence profiles were recorded and fitted for GFP puncta in fluorescence images recorded with settings chosen to avoid saturation of the grey-scale. The standard deviations of the fitted Gaussian curves were used to estimate the true dimensions of the puncta [true radius = SD (puncta image profile) – SD (microsphere image profile) + 85 nm]. GFP contents of puncta were estimated by comparing their fluorescence intensity to the mean fluorescence intensity of patches of cytochrome *bd*-GFP in *E. coli*, which have a mean GFP content of 76 (Lenn *et al*., 2008). Intensity quantification was with ImageJ.

### Electron microscopy

Cultures were harvested by centrifugation, fixed for 2 h at room temperature with 4% (w/v) glutaraldehyde in 100 mM phosphate buffer (pH 7.3) and washed 3× with 100 mM phosphate buffer. After embedding in 2% (w/v) low-gelling-temperature agarose, samples were cut in 1–2 mm cubic blocks, and post-fixed with 2% (w/v) potassium permanganate in distilled water overnight at 4°C. Samples were washed with distilled water until the supernatant remained clear, and dehydrated through a graded ethanol series (1 × 15 min 30%, 1 × 15 min 50%, 1 × 15 min 70%, 1 × 15 min 90% and 3 × 20 min 100%). Two 5 min washes with propylene oxide were performed prior to infiltration with Araldite for 1 h and with fresh Araldite overnight. Polymerisation was achieved by incubation at 60–65°C for 48 h. Thin sections were cut with a glass knife at a Reichert Ultracut E microtome and collected on uncoated 300-mesh copper grids. High contrast was obtained by post-staining with saturated aqueous uranyl acetate and lead citrate (Reynolds, 1963) for 4 min each. The grids were examined in a JEOL JEM-1230 transmission electron microscope at an accelerating potential of 80 kV.

### Oxygen evolution

Oxygen evolution was measured at 30°C in a light-controlled Clarke-type oxygen electrode (Oxylab2, Hansatech, King's Lynn, UK) with saturating red LED illumination at 500 μE m^–2^ s^−1^. LL-grown cells were harvested and resuspended in fresh BG-11 medium to a chlorophyll concentration of 10 μM. Whole-chain oxygen evolution was measured without addition of electron acceptors or inhibitors. Rates measured after HL exposure were corrected for dark respiration and expressed relative to the rate for LL cells.
