# Supplementary material for: Localisation and interactions of the Vipp1 protein in cyanobacteria
Source: Mol Microbiol. 2014 Oct 30;94(5):1179–95. doi: 10.1111/mmi.12826 (PMC4297356; doi:10.1111/mmi.12826)
Supplement: Supplementary file 1 — Supporting information [file mmi0094-1179-sd1.zip › mmi_12826_sup-0001_figureS1-9_tableS1.pdf]

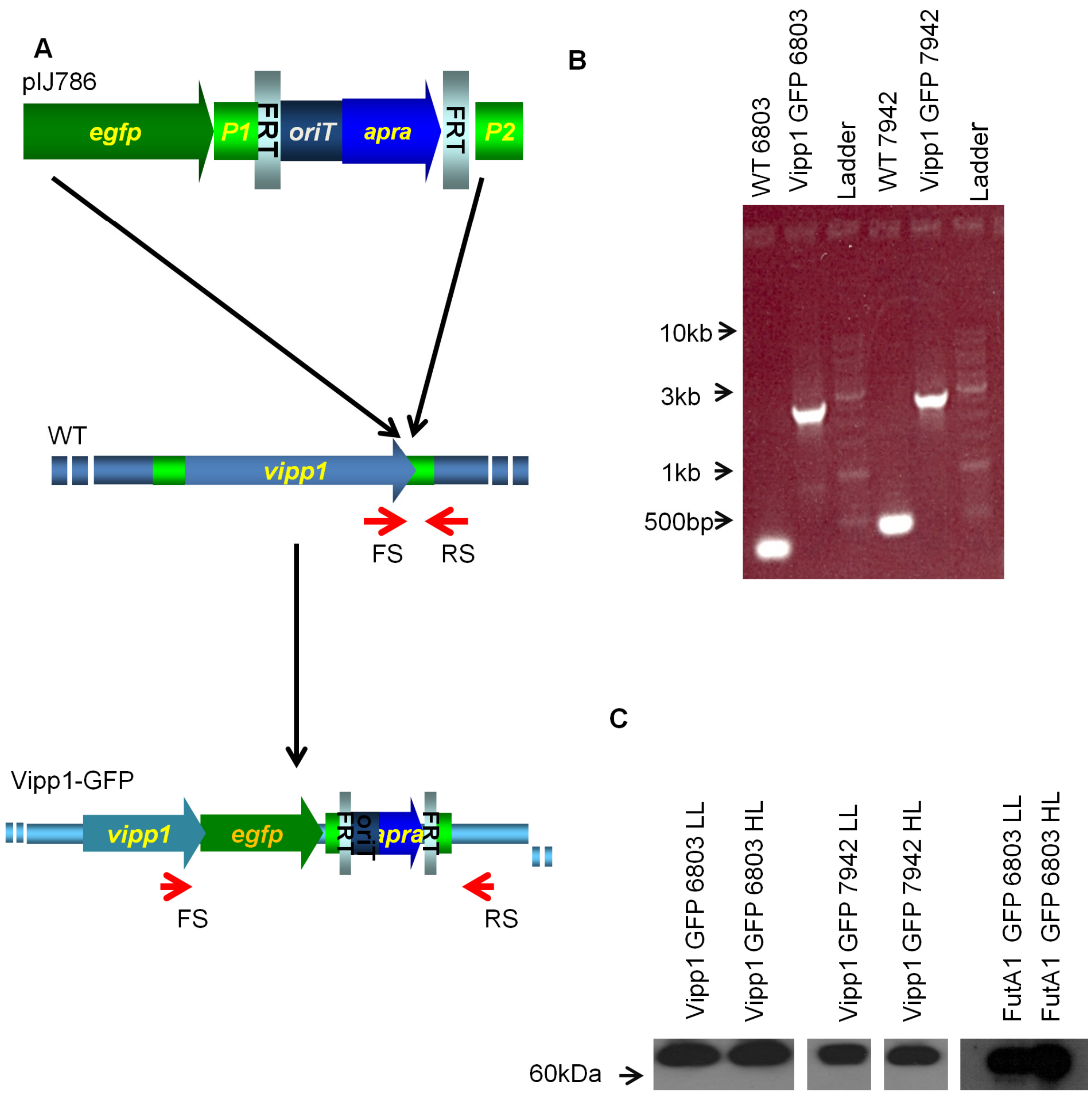

Supporting Figure 1. Construction and genetic and biochemical characterisation of *Synecocystis* and *Synechococcus vipp1-gfp* strains. A. Chromosomal maps of *Synechococcus* and *Synechocystis vipp1-gfp* showing the position of the screening primers 6803 and 7942 FS and RS. **B**. Agarose gels showing full segregation of *Synechocystis* and *Synechococcus vipp1-gfp*. Primers were designed to amplify between the 3' end of the *vipp1* gene and just downstream of *vipp1*. The inclusion of the *gfp* apramycin cassette increases the size of the product to nearly 3kb. **C**. Immunoblots showing undiluted, unfractionated (pre-column) extracts from LL and HL, *Synechocystis* and. *Synechococcus vipp1-gfp* strains and *Synechocystis futA1-gfp* using anti-GFP antibody. Samples are shown from 2 different blots which were run at the same voltage and for the same time and exposed to identical amounts of antibody and ECL. GFP is linked to proteins of the expected size in both *Synechocystis* and. *Synechococcus vipp1-gfp* and for *Synechocystis futA1-gfp*.

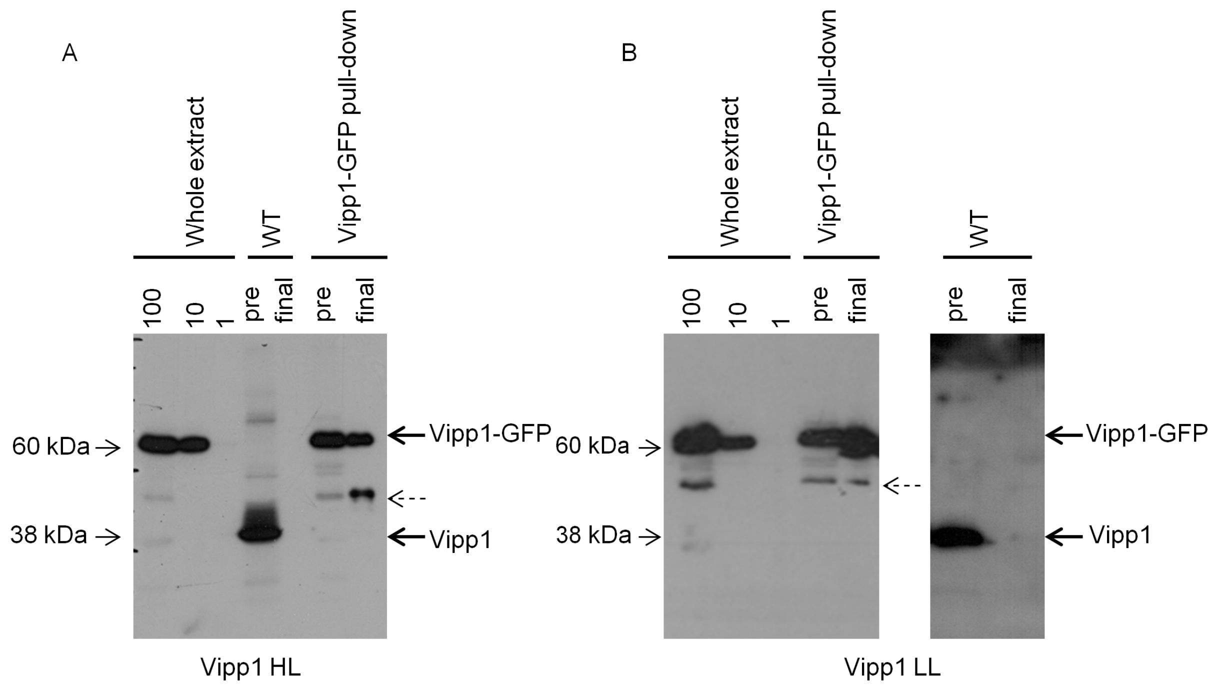

Supporting Figure 2. **Immunoblotting of Vipp1 in *Synechocystis*.**

A: Immunoblot with anti-Vipp1 antibody against extracts from LL *Synechocystis vipp1-gfp* and WT cells. **B:** Immunoblot with anti-Vipp1 antibody against extracts from HL-treated *Synechocystis vipp1-gfp* and WT cells. Samples are shown from 2 different blots which were run at the same voltage and for the same time and exposed to identical amounts of antibody and ECL. Sample dilutions (100%, 10%, and 1%) and undiluted sample for unfractionated (pre-column) *Synechocystis vipp1-gfp* are shown for comparison, whereas the final elution is the fraction retained by GFP-affinity binding. Most Vipp1 is present as the full-length (60 kDa) Vipp1-GFP protein, although a breakdown product (dashed arrow) can be detected. Vipp1 is affinity-bound in both HL and LL *vipp1-gfp* cells, but not in WT.

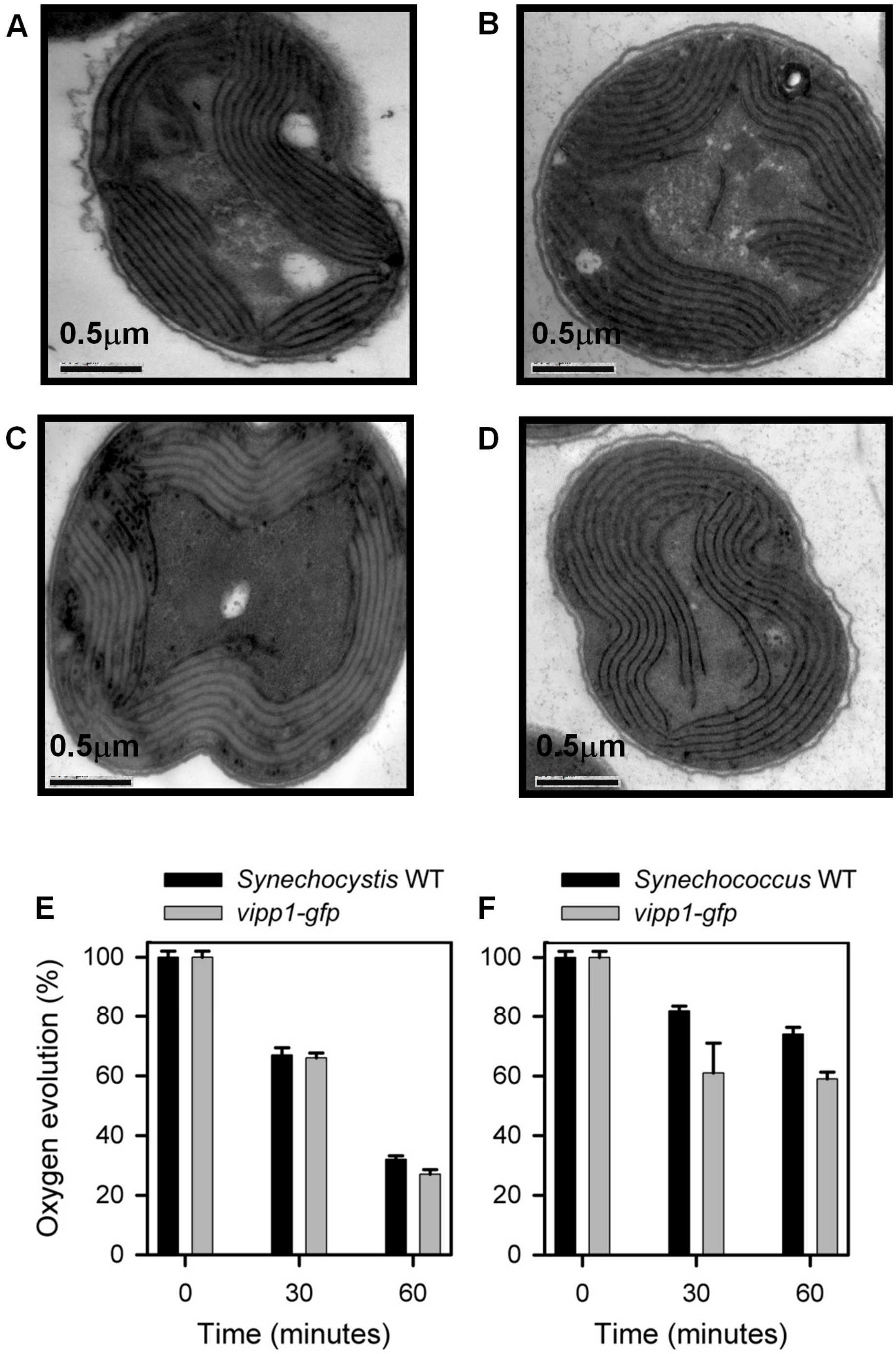

Supporting Figure 3. Phenotypes of *vipp1-gfp* cells in comparison to wild-types. A-D: representative thin-section electron micrographs of *Synechocystis* wild-type cells (**A, B**) and *vipp1-gfp* cells (**C, D**). Cells were grown in low light (**A, C**) or exposed to high light (600 μE m^{-2} s^{-1} , 40 minutes) (**B, D**) prior to fixation. **E**: relative light–saturated oxygen evolution of *Synechocystis* cells following HL exposure (600 μE m^{-2} s^{-1}) for the indicated times. Error bars indicate standard deviations from 3 biological replicates.

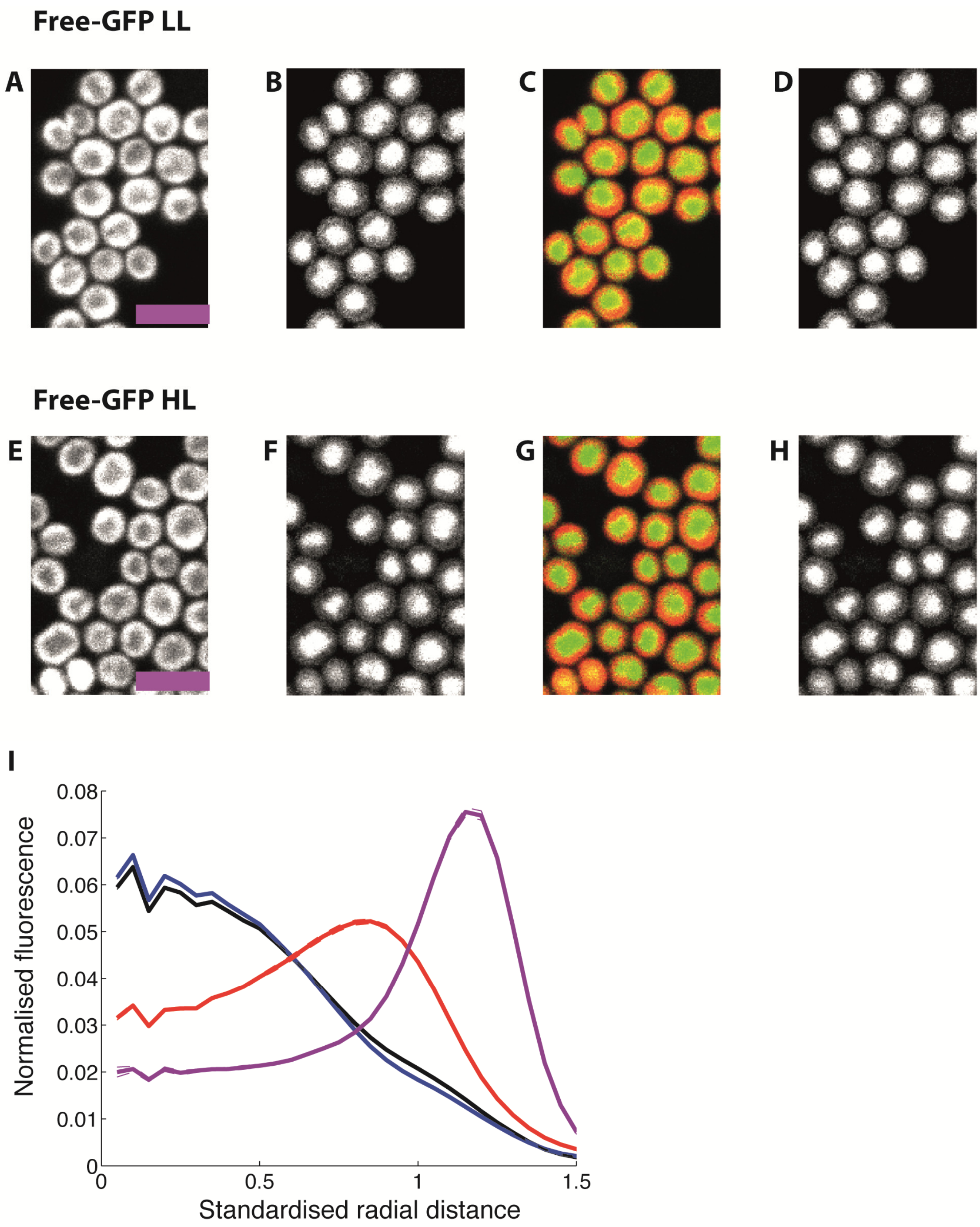

Supporting Figure 4. Free GFP distribution and patterning in *Synechocystis*. Confocal fluorescence micrographs showing chlorophyll fluorescence (first column), GFP (second column), chlorophyll (red):GFP (green) overlay (third column) and located puncta in red (fourth column). *Vipp1-gfp* cells under LL (**A-D**) and after HL exposure for 30 min (**E-H**). **I**. Relative radial distribution of free GFP (black, LL, blue HL) and chlorophyll (red), and FutA-GFP (magenta), a periplasmic protein. S.E.M. are shown as dashed lines, within the width of the line in most cases. Standardised radial distance refers to rescaling the ½ maximum radius for chlorophyll to a radial distance of 1. Scale-bar: 5 microns.

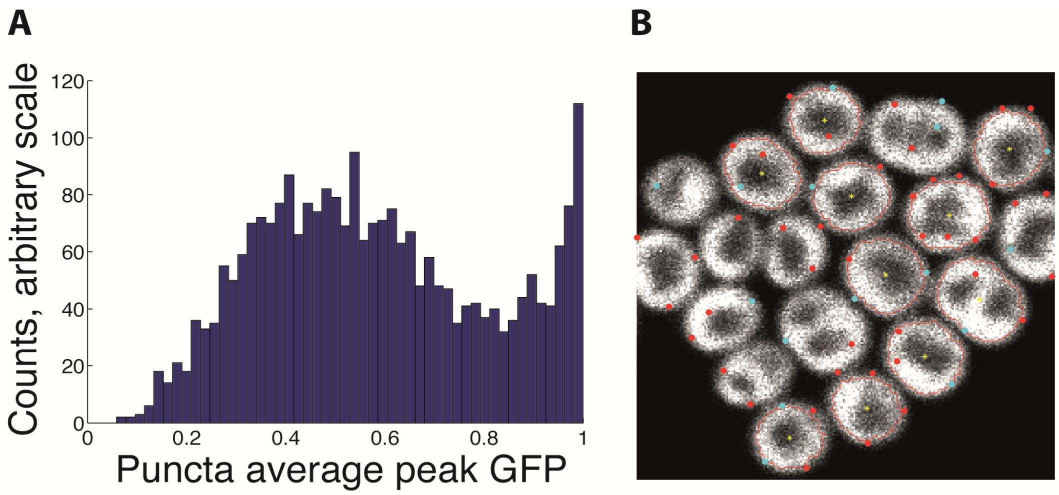

Supporting Figure 5. Heterogeneous populations of Vipp1-GFP puncta in *Synechocystis*.
A: Histogram of peak puncta fluorescence intensity demonstrating brighter (intensity >0.8) and dimmer puncta populations. **B:** Localisation of brighter and dimmer Vipp1-GFP puncta after 80 min. HL exposure, superimposed on the chlorophyll fluorescence image. Bright puncta are shown in cyan, and the others in red.

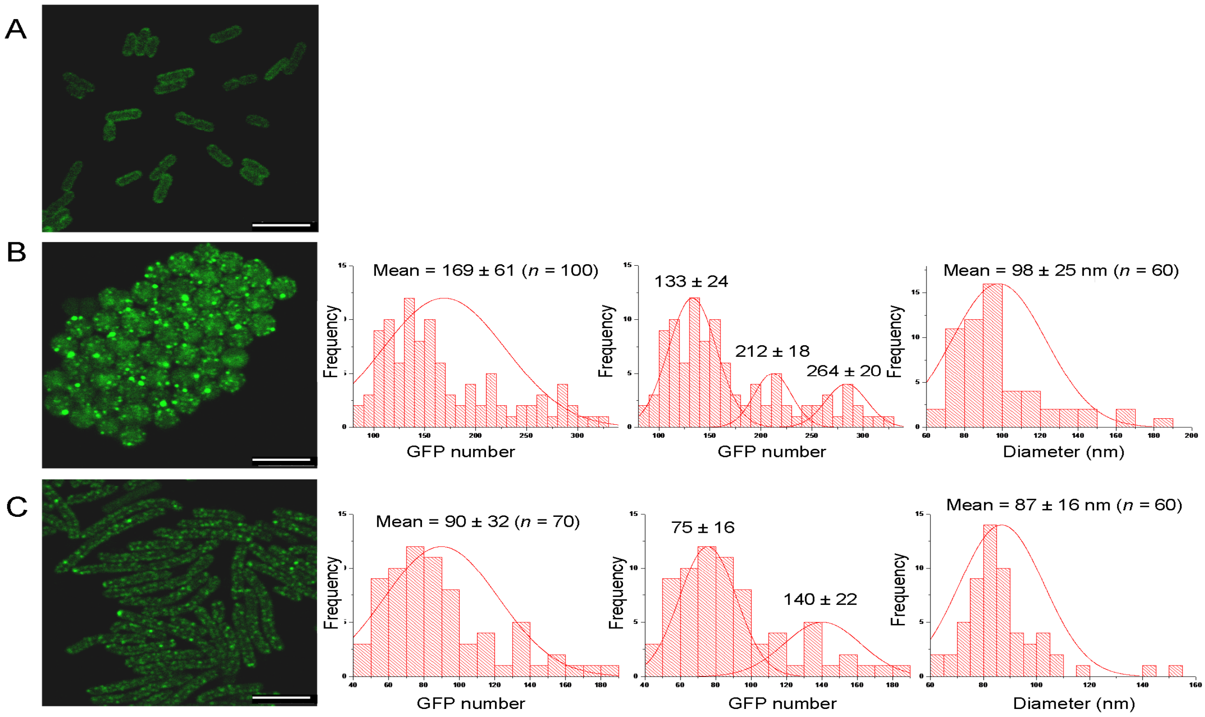

Supporting Figure 6. GFP content and dimensions of high-light induced Vipp1- GFP puncta. A. Confocal fluorescence image of *Escherichia coli* cells used for reference (GFP tag on Cytochrome *bd*: mean GFP content of puncta 76) (Lenn *et al.,* 2008). **B**. *Synechocystis* 6803 *vipp1-gfp*. **C**. *Synechococcus* 7942 *vipp1-gfp*. Scale-bars 5 µm. GFP content estimated from the confocal fluorescence images shown, measuring the brightness of puncta and estimating GFP numbers by comparison with the previously-characterised *Escherichia coli* puncta. All images were recorded with identical microscope settings. Mean GFP contents are calculated on the basis of a single population (left) and 2 or 3 populations (centre). The diameters of the puncta (right) are estimated after correcting for the point-spread function of the microscope, obtained from images of 170 nm diameter fluorescent microspheres recorded with the same microscope settings. Means assume a single population of puncta.

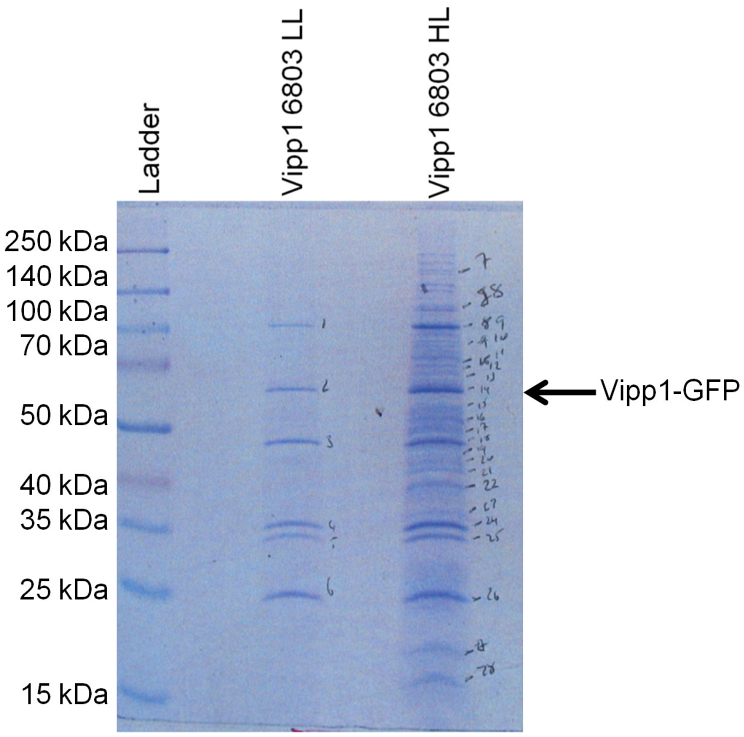

Supporting Figure 7. **Coomassie-stained gel showing the position of the bands excised for mass spectrometry.** Final elutions from the μ-Macs column were separated on a 10% SDS-polyacrylamide gel. Identification of proteins was performed upon in-gel digestion using OMX-S and off-line nanoelectrospray ionization of peptides by *de novo* sequence analysis on a Waters Q-Tof Premier mass spectrometer.

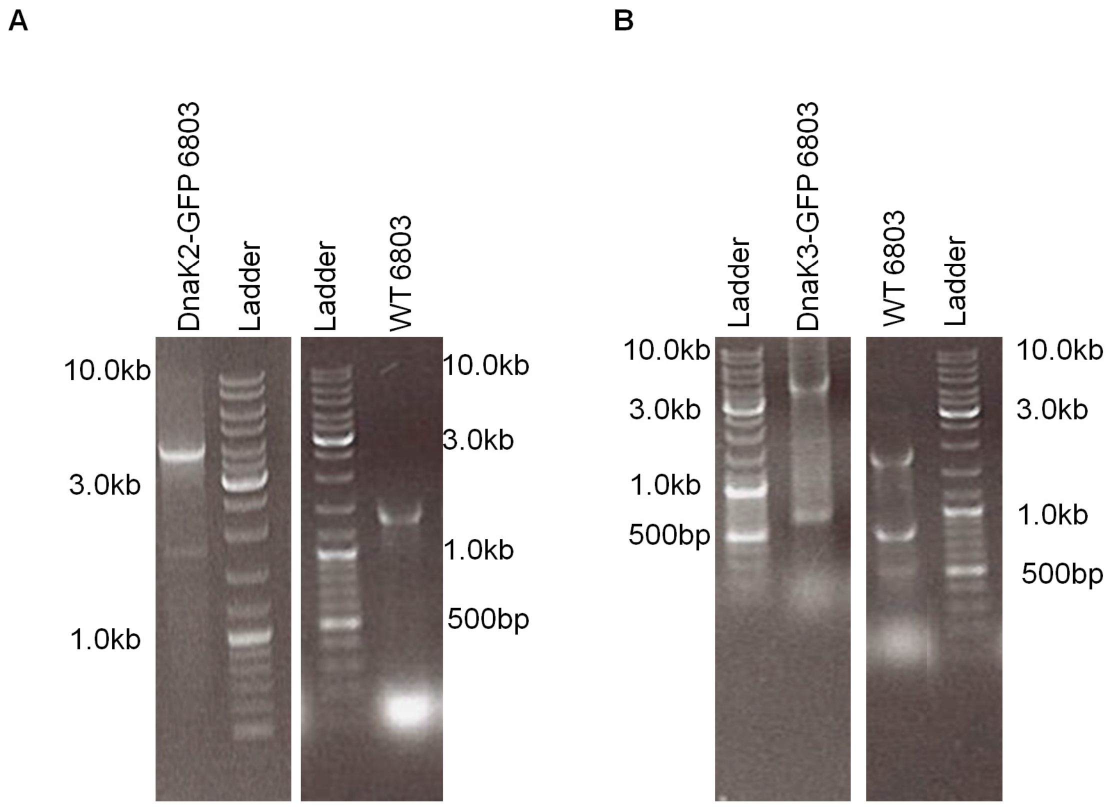

Supporting Figure 8. Genetic characterisation of *Synecocystis dnaK2/3-gfp* strains.

A. Agarose gel showing full segregation of *Synechocystis dnaK2-gfp.* Primers were designed to amplify between the 5' end of the *dnaK2* gene and just downstream of *dnak2*. The inclusion of the *gfp* apramycin cassette increases the size of the product from 1.9kb to ~4.0kb.

B. Agarose gel showing full segregation of *Synechocystis dnaK3-gfp.* Primers were designed to amplify between the 5' end of the *dnaK3* gene and just downstream of *dnak3*. The inclusion of the *gfp* apramycin cassette increases the size of the product from 2.3kb to ~4.4kb. Both *dnaK3-gfp* and WT were run on the same gel using two combs.

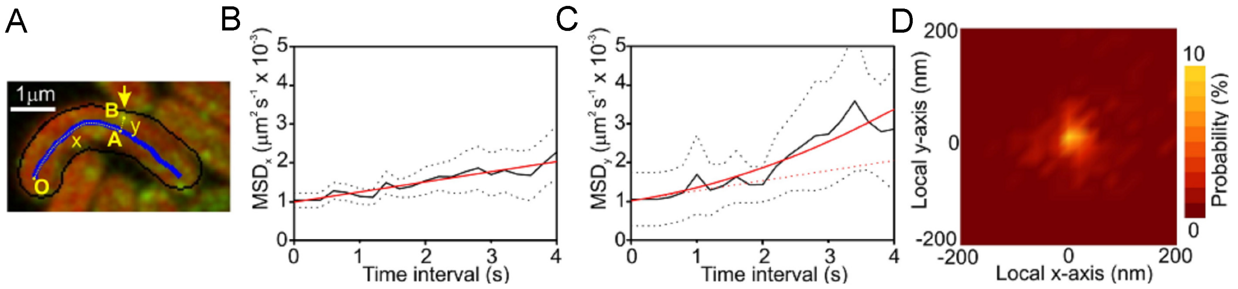

Supporting Figure 9. **Single-particle tracking of Vipp1-GFP puncta in *Synechococcus* from image sequences recorded at 200 ms intervals over a 4s time-window (Supporting Movie 2), tracking 14 particles from different cells. Movement is largely Brownian and isotropic with respect to the local x- and y-axes (respectively the long and short axes of the cells).** **A.** Chlorophyll fluorescence images (red) showing cell outlines (black) and the bisecting long axis (blue). Vipp1-GFP puncta (green) tracked (arrow) to define localisation (point B) to ~20 nm precision. **B.** Average mean-square displacement (black solid line) vs time interval for the local x-axis, with SEM error bounds (dotted lines) and linear fit (red). **C.** As B for the local y-axis, with linear (red dashed) and quadratic (red solid) fits. **D.** Mean spatial probability distribution contour plot of puncta, compiled by re-assigning the first track point as the origin, then overlaying all tracks and re-sampling over 0.5 pixel intervals.

Automated custom-written image analysis software (Xue and Leake, 2009; Xue *et al.*, 2010; Llorente-Garcia Llorente-Garcia, I. *et al.* (2014) *Biochim. Biophys. Acta (BBA Bieenergetics)* **1837**:811-824) was used to define the boundaries of the Vipp1 puncta (Fig. S9A) and to track the puncta, for up to a few seconds duration before puncta either diffused beyond the focus depth of field or other puncta diffused to within the optical resolution limit (~200-300 nm) such that puncta could no longer be distinguished separately. Lateral track coordinates were then converted to a local cellular coordinate system defined by the unique long and short axes of a given cell (Fig. S9A). Each track was converted to mean square displacement (MSD) values as a function of time interval (Xue *et al.*, 2010) and average MSD values were then

compiled for tracks of at least 4 s duration for separate short and long axis coordinates. MSD data for each track could be adequately modelled using a linear fit, indicative of normal Brownian diffusion. Comparison with a quadratic model indicative of directed diffusion showed no significant improvement within the range of the MSD error bounds (Fig. S9B). Mean direction-of-movement probability distributions were generated by re-assigning the first track point in each track as the origin, then overlaying all tracks and re-sampling over 0.5 pixel intervals, and rendering as a probability contour plot (Fig. S9C) indicating broadly isotropic puncta mobility.

Supporting Movie 1. Time-lapse confocal fluorescence image sequences (200 ms intervals) showing the movement of Vipp1-GFP puncta in *Synechocystis* cells. The sequences are presented as avi movie files that play in real time. Excitation was at 488 nm. GFP fluorescence (500-525 nm) is shown in green and chlorophyll fluorescence (670-720 nm, showing the location of the thylakoid membranes) in magenta. The *z*-axis resolution is ~2µm. Cells were pre-treated with white light at 600 µE m^{-2} s^{-1} for 30-60 min to induce Vipp1-GFP puncta formation.

Supporting Movie 2. Time-lapse confocal fluorescence image sequences (200 ms intervals) showing the movement of Vipp1-GFP puncta in *Synechococcus* cells. The sequences are presented as avi movie files that play in real time. Excitation was at 488 nm. GFP fluorescence (500-525 nm) is shown in green and chlorophyll fluorescence (670-720 nm, showing the location of the thylakoid membranes) in magenta. The *z*-axis resolution is ~2µm. Cells were pre-treated with white light at 600 µE m^{-2} s^{-1} for 30-60 min to induce Vipp1-GFP puncta formation.

SUPPORTING TABLE 1

Mass spectrometric identification of proteins after SDS-PAGE separation (see Supporting Fig. 7) in *Synechocystis* sp. PCC 6803 cells exposed to LL and HL

Identified proteins	Gene	Accession number[a]	Detected signals (*m/z*, charge state)	*De novo* sequenced peptides	Sequence coverage (%)
**Synechocystis sp. PCC 6803 - LL**					
Elongation factor Tu<br>**Band 3**	*tuf*	P74227	647.29 $[M + 2 H]^{2+}$<br>758.49 $[M + 2 H]^{2+}$<br>844.44 $[M + 2 H]^{2+}$<br>852.45 $[M + 2 H]^{2+}$<br>1168.11 $[M + 2 H]^{2+}$	AVDDYIDTPER<br>EEISIVGIKDTR<br>EEGMAGDNVGLLLR<br>TLEEGMAGDNVGLLLR<br>DFPGDDIPIVAG	13
Green fluorescent protein **Band 6**	*GFP*	P42212	641.79 $[M + 2 H]^{2+}$	SAMPEGYVQER	5
Phycobiliprotein ApcE<br>**Band 1**	*apcE*	Q55544	604.82 $[M + 2 H]^{2+}$<br>889.36 $[M + 2 H]^{2+}$<br>985.04 $[M + 2 H]^{2+}$	FGESSTQALIR<br>EYSDAFGEDTVPYER<br>DLEPYIINSEFTALESK	5
Phycobilisome 32.1 kDa linker polypeptide, phycocyanin-associated, rod 1<br>**Band 4**	*cpcC1*	P73203	601.80 $[M + 2 H]^{2+}$<br>616.85 $[M + 2 H]^{2+}$<br>673.37 $[M + 2 H]^{2+}$<br>702.29 $[M + 2 H]^{2+}$<br>770.36 $[M + 2 H]^{2+}$<br>778.39 $[M + 2 H]^{2+}$	FLYNNFQTR<br>ALGGTVPFGQASK<br>VEITAISAPGYPK<br>GAESLLTNGSISVR<br>QVLGNDYIMDSER<br>QVLGNDYIMDSER	21
Phycobilisome 32.1 kDa linker polypeptide, phycocyanin-associated, rod 2 **Band 5**	*cpcC2*	P73204	804.48 $[M + 2 H]^{2+}$<br>847.45 $[M + 2 H]^{2+}$	IVAVDEAIPLELR<br>STEEEVDAVILAVYR	10
Plasma membrane protein essential for thylakoid formation **Band 2**	*vipp1*	F7USL8	650.85 $[M + 2 H]^{2+}$<br>933.48 $[M + 2 H]^{2+}$<br>1376.00 $[M + 3 H]^{3+}$	LALTNGEENLAR<br>TDTAAAYQTQLAQQR<br>GELAGFGIENQFAQLEASSGVEDELAALK	21
**Synechocystis sp. PCC 6803 - HL**					
Aconitate hydratase 2 **Band 14**	*acnB*	P74582	946.46 $[M + 2 H]^{2+}$	YLNFNEIDNFEDFGR	2
Allophycocyanin alpha chain<br>**Band 27**	*apcA*	Q01951	523.24 $[M + 2 H]^{2+}$<br>525.29 $[M + 2 H]^{2+}$<br>714.42 $[M + 2 H]^{2+}$<br>791.41 $[M + 2 H]^{2+}$<br>1186.62 $[M + 2 H]^{2+}$	VNADAEAR<br>YLSPGELDR<br>SLGTPIEAVAQSVR<br>TPIEEIGLVGVR<br>VTYGVVSGDVTPIEEIGLVGVR	33
Argininosuccinate synthase **Band 17**	*argG*	P77973	1005.00 $[M + 2 H]^{2+}$	AIADTPDEPEYVDIGFEK	5

Aspartyl/glutamyl-tRNA(Asn/Gln) amidotransferase subunit B **Band 16**	*gatB*	P74215	747.56 $[M + 2 H]^{2+}$	ADGEEIYQETR	2
ATP synthase subunit beta **Band 17**	*atpD*	P26527	1058.03 $[M + 2 H]^{2+}$	VDTGAPISVPVGTGTLGR	4
C-phycocyanin alpha chain<br>**Band 28**	*cpcA*	Q54715	734.93 $[M + 2 H]^{2+}$<br>766.88 $[M + 2 H]^{2+}$<br>828.42 $[M + 2 H]^{2+}$<br>879.71 $[M + 3 H]^{3+}$<br>904.46 $[M + 2 H]^{2+}$<br>1319.10 $[M + 2 H]^{2+}$	FLSSTELQIAFGR<br>TPLTEAVSTADSQGR<br>TFDLSPSWYVEALK<br>ANHGLSGDARDEANSYLDYAINALS<br>MKTPLTEAVSTADSQGR<br>EANSYLDYAINALS	43
C-phycocyanin beta chain<br>**Band 27**	*cpcB*	Q54714	458.23 $[M + 2 H]^{2+}$<br>672.87 $[M + 2 H]^{2+}$<br>677.01 $[M + 3 H]^{3+}$<br>773.90 $[M + 4 H]^{4+}$<br>799.41 $[M + 2 H]^{2+}$<br>954.54 $[M + 2 H]^{2+}$<br>979.51 $[M + 3 H]^{3+}$<br>1031.48 $[M + 2 H]^{2+}$<br>1055.50 $[M + 2 H]^{2+}$	MFDVFTR<br>ITGNASAIVSNAAR<br>IDSVNRITGNASAIVSNAAR<br>VVSQADARGEYLSGSQLDALSATVAEGNKR<br>EAALDIVNDPNGITR<br>YVTYATFTGDASVLEDR<br>VSQADARGEYLSGSQLDALSATVAEGNK<br>VSQADARGEYLSGSQLDALSATVAEGNKR<br>YLSGSQLDALSATVAEG	52
Chaperone protein dnaK2<br>**Band 12**	*dnaK2*	P22358	683.33 $[M + 2 H]^{2+}$<br>708.86 $[M + 2 H]^{2+}$<br>744.35 $[M + 2 H]^{2+}$<br>1067.51 $[M + 3 H]^{3+}$	NQADSLVYQAEK<br>SALDEIVLVGGSTR<br>QFAPEEISAQVLR<br>AVITVPAYFNDSQR	8
Chaperone protein dnaK3 **Band 9**	*dnaK3*	P73098	991.53 $[M + 2 H]^{2+}$	DAGLSPVQIDEVVLVGGGTR	3
Elongation factor G 1 **Band 8**	*fusA*	P28371	1021.83 $[M + 3 H]^{3+}$<br>1050.01 $[M + 2 H]^{2+}$	YAIVDPEAPLR<br>IDYIVEPGEPGSGFQFESK	4
Elongation factor Tu<br>**Band 19**	*tuf*	P74227	468.16 $[M + 2 H]^{2+}$<br>544.31 $[M + 2 H]^{2+}$<br>647.29 $[M + 2 H]^{2+}$<br>779.01 $[M + 3 H]^{3+}$<br>844.44 $[M + 2 H]^{2+}$<br>890.01 $[M + 2 H]^{2+}$<br>903.44 $[M + 2 H]^{2+}$<br>908.00 $[M + 2 H]^{2+}$<br>916.01 $[M + 2 H]^{2+}$<br>1023.53 $[M + 2 H]^{2+}$	TTDVTGTIK<br>GIQKEDIER<br>AVDDYIDTPER<br>PGDDIPIVAGSALK<br>TLEEGMAGDNVGLLLR<br>TTLTAAITMTLAELGGAK<br>GITINTAHVEYETDSR<br>MTVELINPIAIEQGMR<br>MTVELINPIAIEQGMR<br>DMVDDEELLELVELEVR	34

			1031.49 $[M + 2 H]^{2+}$<br>1168.10 $[M + 2 H]^{2+}$	DMVDDEELLELVELEVR<br>ELLSDYDFPGDDIPIVAGSALK	
Ferredoxin--NADP reductase **Band 17**	*petH*	Q55318	1050.53 $[M + 2 H]^{2+}$	EGAIGTVQHLTFDLSAGDLR	5
Glycine--tRNA ligase beta subunit **Band 9**	*glyS*	Q55690	1161.58 $[M + 2 H]^{2+}$	PLQTFLFEIGTEELPADFVR	3
Green fluorescent protein **Band 15**	*GFP*	P42212	641.79 $[M + 2 H]^{2+}$<br>752.33 $[M + 2 H]^{2+}$<br>813.09 $[M + 3 H]^{3+}$	SAMPEGYVQER<br>FSVSGEGEGDATYGK<br>GEELFTGVVPILVELDGDVNGHK	21
GTP-binding protein TypA/BipA homolog **Band 13**	*typA*	P72749	914.37 $[M + 2 H]^{2+}$<br>1072.54 $[M + 2 H]^{2+}$<br>1116.53 $[M + 2 H]^{2+}$	EFQVAQPQVIYR<br>ALEYIGPDELVEITPESIR<br>VQLQAPEDMNLER	7
Light-dependent protochlorophyllide reductase **Band 22**	*por*	Q59987	883.30 $[M + 2 H]^{2+}$	EAFVQELSEQGSDAQK	5
Light repressed protein A homolog **Band 26**	*lrtA*	P74518	555.32 $[M + 2 H]^{2+}$<br>958.45 $[M + 3 H]^{3+}$<br>1021.88 $[M + 2 H]^{2+}$<br>1045.55 $[M + 3 H]^{3+}$	APELPSEVLR<br>GSENLYASIDLVADK<br>TSEIVEDKPVEENLIGDR<br>TSEIVEDKPVEENLIGDRAPELPSEVLR	23
Putative peroxiredoxin sll1621 **Band 28**	*sll1621*	P73728	570.84 $[M + 2 H]^{2+}$<br>609.77 $[M + 2 H]^{2+}$<br>757.88 $[M + 2 H]^{2+}$	APGVSEPVK<br>PDGNGEFTR<br>EAPGVSEPVKAFV	12
Orange carotenoid-binding protein **Band 22**	*slr1963*	P74102	740.30 $[M + 2 H]^{2+}$<br>746.28 $[M + 2 H]^{2+}$<br>993.89 $[M + 2 H]^{2+}$	EPVVPPQDTASR<br>TEPAEDGFTQIK<br>IAAPGAASMQLAENALK	13
Photoreceptor for positive phototaxis PixJ1 **Band 10**	*pixJ1*	Q54A85	821.87 $[M + 2 H]^{2+}$<br>870.41 $[M + 3 H]^{3+}$<br>922.50 $[M + 2 H]^{2+}$	LSDIDEIQGVIQK<br>SQFTETTDTNEVAVR<br>SIQAVAENAAQAESAVQR	5
Photosystem I P700 chlorophyll a apoprotein A1 **Band 18**	*psaA*	P29254	766.91 $[M + 2 H]^{2+}$	VSVDNNPVPTSFEK	2
Phosphoglycerate kinase **Band 20**	*pgk*	P74421	570.32 $[M + 2 H]^{2+}$<br>819.01 $[M + 2 H]^{2+}$	VLPGIAALDDR<br>IASLPNGGVALLENLR	7
Phosphoribulokinase **Band 21**	*prk*	P37101	928.01 $[M + 2 H]^{2+}$	ELVDFGVYLDISEEVK	5
Phycobiliprotein ApcE **Band 8**	*apcE*	Q55544	594.34 $[M + 2 H]^{2+}$<br>604.82 $[M + 2 H]^{2+}$<br>642.83 $[M + 2 H]^{2+}$	ALVGASSDSR<br>FGESSTQALIR<br>QFFEPFINSR	29

			726.73 [M + 3 H]$^{3+}$	FVELGQVSAIRTEPEIAYR	
			787.43 [M + 2 H]$^{2+}$	FPTLPAA	
			815.91 [M + 2 H]$^{2+}$	AYSQSISYLESQVR	
			866.99 [M + 2 H]$^{2+}$	EIQQYNQILASQGLK	
			871.97 [M + 2 H]$^{2+}$	LSVAEIQLENGDISVR	
			889.36 [M + 2 H]$^{2+}$	SDAFGEDTVPYER	
			943.55 [M + 2 H]$^{2+}$	LEIAETLTQNADLIVSR	
			971.98 [M + 2 H]$^{2+}$	NGSVTYAESNGSGGLFGGLR	
			985.04 [M + 2 H]$^{2+}$	DLEPYIINSEFTALESK	
			1015.45 [M + 3 H]$^{3+}$	LARPQLYQTVPVSAISQAEQQDR	
			1089.58 [M + 2 H]$^{2+}$	IRTEPEIAYR	
			1134.10 [M + 2 H]$^{2+}$	GPAVNNQVGNPSAVGEFPGSLGAK	
			1174.61 [M + 2 H]$^{2+}$	QGPSNDIQGLELPQSYFNAAAK	
			1528.79 [M + 3 H]$^{3+}$	LVDALVDSQEYADYFGEETVPY	
Phycobilisome 32.1 kDa linker polypeptide, phycocyanin-associated, rod 1<br>**Band 23**	*cpcC1*	P73203	475.17 [M + 2 H]$^{2+}$	FNNQVGDR	41
			601.80 [M + 2 H]$^{2+}$	FLYNNFQTR	
			616.85 [M + 2 H]$^{2+}$	ALGGTVPFGQASK	
			673.37 [M + 2 H]$^{2+}$	ITAISAPGYPK	
			702.29 [M + 2 H]$^{2+}$	AESLLTNGSISVR	
			704.32 [M + 3 H]$^{3+}$	VPFEQLNQTLQQINR	
			770.36 [M + 2 H]$^{2+}$	QVLGNDYIMDSER	
			778.39 [M + 2 H]$^{2+}$	LGNDYIMDSER	
			1055.94 [M + 2 H]$^{2+}$	AVIVPFEQLNQTLQQINR	
			1367.45 [M + 4 H]$^{4+}$	LYENQGFDADIDSYIDSVEYQENFGENIVPYYR	
			1822.73 [M + 3 H]$^{3+}$	GFDADIDSYIDSVEYQENFGENIVPYYR	
Phycobilisome 32.1 kDa linker polypeptide, phycocyanin-associated, rod 2<br>**Band 24**	*cpcC2*	P73204	495.28 [M + 2 H]$^{2+}$	LTSAESLLR	30
			555.32 [M + 2 H]$^{2+}$	LQVIQGAAPGR	
			565.31 [M + 3 H]$^{3+}$	STEEEVDAVILAVYR	
			736.87 [M + 2 H]$^{2+}$	YLVSYDNLSAK	
			804.48 [M + 2 H]$^{2+}$	LGIVAVDEAIPLELR	
			812.41 [M + 2 H]$^{2+}$	NTASPVYAGSTAESLR	
			829.42 [M + 2 H]$^{2+}$	GKAEYLVSYDNLSAK	
			847.45 [M + 2 H]$^{2+}$	STEEEVDAVILAVYR	
Phytoene dehydrogenase **Band 16**	*pds*	P29273	776.01 [M + 2 H]$^{2+}$	YLADAGFTPVVLER	3
Plasma membrane protein essential for	*vipp1*	F7USL8	650.85 [M + 2 H]$^{2+}$	LALTNGEENLAR	37

thylakoid formation<br>**Band 15**			770.36 $[M + 3 H]^{3+}$<br>890.13 $[M + 3 H]^{3+}$<br>895.46 $[M + 3 H]^{3+}$<br>933.48 $[M + 2 H]^{2+}$<br>1000.03 $[M + 2 H]^{2+}$<br>1008.03 $[M + 2 H]^{2+}$<br>1028.50 $[M + 4 H]^{4+}$<br>1032.23 $[M + 4 H]^{4+}$<br>1155.09 $[M + 2 H]^{2+}$<br>1370.68 $[M + 3 H]^{3+}$<br>1376.00 $[M + 3 H]^{3+}$	LQQTLGGLGTSSATSAFER<br>VIDMQEDLVQLR<br>QAVIDMQEDLVQLR<br>SLTDTAAAYQTQLAQQR<br>EQAVIDMQEDLVQLR<br>VLEQAVIDMQEDLVQLR<br>ELAGFGIENQFAQLEASSGVEDELAALK<br>FAQLEASSGVEDELAALK<br>ANAELQQTLGGLGTSSATSAFER<br>AGELAGFGIENQFAQLEASSGVEDELAALK<br>AGFGIENQFAQLEASSGVEDELAALK	
Polyphosphate kinase **Band 11**	*ppk*	Q55898	1113.53 $[M + 2 H]^{2+}$	GIFLNDYVDLSQEER	2
Polyribonucleotide nucleotidyltransferase **Band 9**	*pnp*	P72659	839.95 $[M + 2 H]^{2+}$<br>977.05 $[M + 2 H]^{2+}$	VGLVGDDFIINPTYR<br>IPVLPPQEDFPYVVR	4
Protein translocase subunit SecA<br>**Band 7**	*secA*	Q55709	683.37 $[M + 2 H]^{2+}$<br>799.89 $[M + 2 H]^{2+}$<br>1173.60 $[M + 2 H]^{2+}$	FFLSLEDNLLR<br>GTDIILGGNSDYMAR<br>SDAETEEILDEILPEAFAVVR	5
(P)ppGpp 3'-pyrophosphohydrolase<br>**Band 10**	*spoT*	F7UQ39	687.41 $[M + 2 H]^{2+}$	KDIFAPLANR	1
Ribonuclease II **Band 12**	*rnb*	F7UM89	742.82 $[M + 2 H]^{2+}$<br>966.29 $[M + 3 H]^{3+}$	LLSPNDELDLEAR<br>TYTIDDESTSEIDDGLSVETLADGGHR	6
Ribulose bisphosphate carboxylase large chain **Band 18**	*cbbL*	P54205	612.32 $[M + 2 H]^{2+}$<br>736.84 $[M + 2 H]^{2+}$<br>1320.58 $[M + 3 H]^{3+}$	FLFVQEAIEK<br>EIKFEFEAMDTL<br>AVAAESSTGTWTTVWTDNLTDLDR	10
Thiol-specific antioxidant protein<br>**Band 25**	*sll0755*	F7US08	980.02 $[M + 2 H]^{2+}$	SQAYNVLEPDAGIA	7
Transketolase **Band 11**	*tktA*	P73282	864.89 $[M + 2 H]^{2+}$	VVATQSLDELSINAIR	2
Translation initiation factor IF-2<br>**Band 10**	*infB*	P72689	551.82 $[M + 2 H]^{2+}$<br>624.36 $[M + 2 H]^{2+}$<br>847.43 $[M + 2 H]^{2+}$<br>866.48 $[M + 2 H]^{2+}$<br>1027.12 $[M + 2 H]^{2+}$	PPQPPVAK<br>DAIVVGAVYGK<br>MVAESFEVAVETPER<br>AVQITQTLDEETAR<br>ILVVAADDGVQPQT	6
Uncharacterized sugar kinase slr0537<br>**Band 22**	*slr0537*	Q55480	980.38 $[M + 2 H]^{2+}$	GGAGSLIFDGENLLTIGTPK	6

60 kDa chaperonin 1<br>**Band 15**	*groEL1*	Q05972	685.69 $[M + 3H]^{3+}$<br>764.98 $[M + 2H]^{2+}$<br>1192.61 $[M + 3H]^{3+}$	TTATVLAHAIVK<br>IAENAGQNGAVISER<br>QMLEDIATLTGGQVISEDAGLKLESATVDSLGSAR	12

[a] UniProtKB/SwissProt database entry number from *Synechocystis* sp. PCC6803
